# Visible Light-Induced Homolytic Cleavage of Perfluoroalkyl Iodides Mediated by Phosphines

**DOI:** 10.3390/molecules25071606

**Published:** 2020-04-01

**Authors:** Mario Bracker, Lucas Helmecke, Martin Kleinschmidt, Constantin Czekelius, Christel M. Marian

**Affiliations:** 1 Institute of Theoretical and Computational Chemistry, Heinrich-Heine-University Düsseldorf, 40204 Düsseldorf, Germany; mario.bracker@hhu.de; 2 Institute of Organic and Macromolecular Chemistry, Heinrich-Heine-University Düsseldorf, 40204 Düsseldorf, Germany; lucas.helmecke@hhu.de

**Keywords:** halogen bond, iodoperfluoroalkylation, radical, absorption spectra, adiabatic transition, intersystem crossing, density functional thory, multireference configuration interaction, spin–orbit coupling

## Abstract

In an effort to explain the experimentally observed variation of the photocatalytic activity of ${}^{t}$Bu${}_{3}$P, ${}^{n}$Bu${}_{3}$P and (MeO)${}_{3}$P in the blue-light regime [Helmecke et al., Org. Lett. 21 (2019) 7823], we have explored the absorption characteristics of several phosphine– and phosphite–IC${}_{4}$F${}_{9}$ adducts by means of relativistic density functional theory and multireference configuration interaction methods. Based on the results of these computational and complementary experimental studies, we offer an explanation for the broad tailing of the absorption of ${}^{t}$Bu${}_{3}$P-IC${}_{4}$F${}_{9}$ and (MeO)${}_{3}$P-IC${}_{4}$F${}_{9}$ into the visible-light region. Larger coordinate displacements of the ground and excited singlet potential energy wells in ${}^{n}$Bu${}_{3}$P-IC${}_{4}$F${}_{9}$, in particular with regard to the P–I–C bending angle, reduce the Franck–Condon factors and thus the absorption probability compared to ${}^{t}$Bu${}_{3}$P-IC${}_{4}$F${}_{9}$. Spectroscopic and computational evaluation of conformationally flexible and locked phosphites suggests that the reactivity of (MeO)${}_{3}$P may be the result of oxygen lone-pair participation and concomitant broadening of absorption. The proposed mechanism for the phosphine-catalyzed homolytic C–I cleavage of perfluorobutane iodide involves S_1_ ← S_0_ absorption of the adduct followed by intersystem crossing to the photochemically active T${}_{1}$ state.

## 1. Introduction

Organic compounds incorporating fluorine substituents or a perfluoroalkyl group show unique properties and are therefore very important synthetic targets in pharmaceutical research and industry [1,2,3,4]. The introduction of fluorine as the most electronegative element results in a different polarization profile of the molecule associated with improved pharmacodynamic and -kinetic properties. It is therefore not surprising that the development of a synthetic methodology for the efficient, safe, and environmentally benign preparation of these compounds by transition metal catalysis or photocatalysis has found increasing interest in the last decades [5,6,7,8,9,10].

The introduction of perfluoroalkyl groups or perfluoroalkanoates by addition of the corresponding perfluoroalkyl iodide to unsaturated hydrocarbons such as alkenes or alkynes is synthetically highly valuable since a plethora of such starting materials are easily accessible and commercially available. In addition, subsequent displacement of the iodine substituent by nucleophilic substitution or metalation allows for further functionalization of the fluorinated molecule. The iodoperfluoroalkylations of double bonds can be initiated by homolytic C–I bond cleavage using radical initiators [11,12] or UV irradiation [13,14]. In cases where such conditions are not advisable for delicate substrates, photocatalytic methods [15,16,17] using visible light irradiation offers advantages. Herein, transition metal complexes based on iridium or ruthenium [18,19], as well as copper [20,21,22], have been successfully employed for atom transfer radical addition (ATRA) reactions.

In addition to metal complexes, organic photocatalysts have also been reported for iodoperfluoroalkylation reactions of unsaturated hydrocarbons, either by using them as triplet sensitizers [23,24] or by activation via the corresponding electron donor–acceptor (EDA) complexes [25,26,27]. In the latter case, the formation of a halogen bond between the electron-deficient perfluoroalkyl iodide and a suitable Lewis base results in homolytic bond cleavage upon irradiation of visible light. Amines [28,29,30,31,32,33], phenols [34], ketones [35,36], as well as phosphines [37,38,39,40,41] have been reported for this purpose in either stoichiometric or catalytic quantities.

**Scheme 1:**
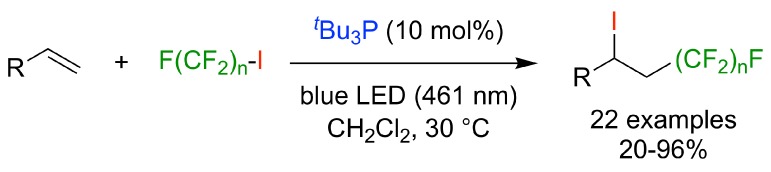
Phosphine-catalyzed iodoperfluoroalkylation of simple alkenes.

In our studies addressing the Lewis-base-mediated iodoperfluoroalkylation of simple alkenes [40,42,43] we found that catalytic amounts of phosphines and phosphites effectively catalyze this process with complete regioselectivity upon irradiation with visible light (461 nm) (Scheme 1). Mechanistic investigations involving ${}^{19}$F-NMR analysis suggested the intermediate formation of an EDA complex F(CF${}_{2}$)_n_–I⋯PR${}_{3}$ by halogen bond formation to the phosphorus atom [44,45]. In addition, the reaction was shown to proceed via free perfluoroalkyl radicals. It is therefore assumed that visible light absorption of the EDA complex formation leads to homolytic bond cleavage and subsequent radical chain reaction.

**Figure 1:**
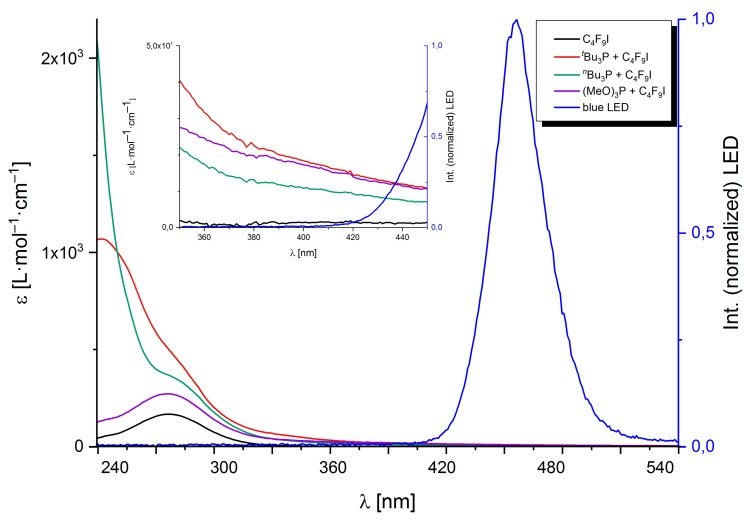
UV-vis spectra of C${}_{4}$F${}_{9}$I, ${}^{t}$Bu${}_{3}$P, ${}^{n}$Bu${}_{3}$P, (MeO)${}_{3}$P and mixtures of the phosphorus compounds with C${}_{4}$F${}_{9}$I.

Two observations called for a more detailed analysis, however. First, among the numerous tested phosphorus compounds, tri(*tert*-butyl)phosphine (${}^{t}$Bu${}_{3}$P) showed the fastest conversion, while tri(*n*-butyl)phosphine (${}^{n}$Bu${}_{3}$P) with comparable lone pair donor properties was much less efficient. In contrast, electron-deficient trimethylphosphite ((MeO)${}_{3}$P) performed very well. Second, the UV-vis spectra of EDA complexes formed upon mixing perfluorobutyl iodide (C${}_{4}$F${}_{9}$I) with the above-mentioned phosphorus (III) compounds—without an alkene present—showed increased absorption but substantially different peak tailing into the visible light region [46]. In addition, in all cases the overlap of the EDA complex absorption with the emission spectrum of the blue LED employed in the reaction ($\lambda_{max}$ = 461 nm) was very small (Figure 1). As a result, the different catalyst performances cannot be attributed exclusively to the absorption profile or the donor properties, but additional factors such as the geometry-dependency of the corresponding singlet–triplet transitions need to be taken into account. Therefore, we aimed at an improved understanding of the photochemical, homolytic bond cleavage process by calculation of the excited-state geometries of the perfluorobutyl iodide adducts [47,48,49] as well as a modeling of the intersystem crossing (ISC) process.

## 2. Results

### 2.1. Quantum Chemical Characterization of the Compounds in the Franck–Condon Region

#### 2.1.1. Perfluorobutyl Iodide

**Figure 2:**
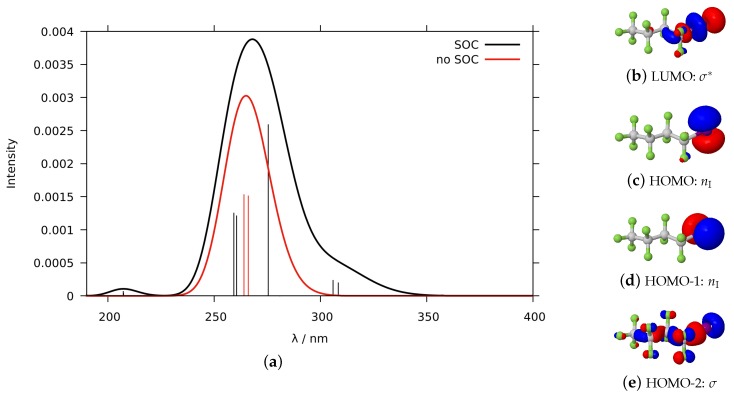
Calculated first absorption band of C${}_{4}$F${}_{9}$I (red: no spin–orbit coupling, only singlet transitions, black: including spin–orbit coupling, singlet as well as triplet transitions) and molecular orbitals involved in the transitions. The spectral envelope was obtained by broadening the line spectrum by Gaussians with standard deviation σ=1500 cm${}^{-1}$. HOMO denotes the highest occupied molecular orbital, LUMO the lowest unoccupied molecular orbital.

Due to the presence of the heavy iodine atom, relativistic calculations have to be performed for modeling the photophysics and photochemistry of the compounds. Spin selection rules are therefore not strictly obeyed. Spin–orbit interaction is particularly pronounced between electronic states involving differently oriented *p*-type orbitals, located at the iodine center, such as the lone-pair $n_{I}$ orbitals that represent the nearly degenerate highest occupied molecular orbital (HOMO) and HOMO-1 in the C${}_{4}$F${}_{9}$I ground-state geometry. The C–I σ bonding (HOMO-2) and $\sigma^{*}$ antibonding (lowest unoccupied molecular orbital, LUMO) involve iodine *p* atomic orbitals as well (Figure 2).

The vertical absorption spectrum of the non-coordinated perfluorobutyl iodide molecule is characterized by weak $\left(n_{I}\to \sigma^{*}\right)$ transitions in the middle-UV region and a strong $\left(\sigma \to \sigma^{*}\right)$ transition in the far UV (Figure S1). With regard to the current experiments, only the weak first absorption band is of interest. The spectral broadening and the enhancement of that band, which are observed when spin–orbit coupling (SOC) is switched on (Figure 2a), can be traced back to intensity borrowing of the spin-forbidden ${}^{3}\left(n_{I}\sigma^{*}\right)$ transitions from the optically bright ${}^{1}\left(\sigma \sigma^{*}\right)$ transition at 167 nm. Comparison between theory and experiment (Figure 2a and Figure S2) reveals excellent agreement of the spectral shapes and absorption maxima $\lambda_{max}$ (theory: 268 nm, experiment: 270 nm).

We observe a small red shift of the absorption maximum by 6 nm when basis sets of higher quality are employed, i.e., triple zeta plus polarization functions on all atoms (cf. Figure S3).

#### 2.1.2. Phosphines

**Figure 3:**
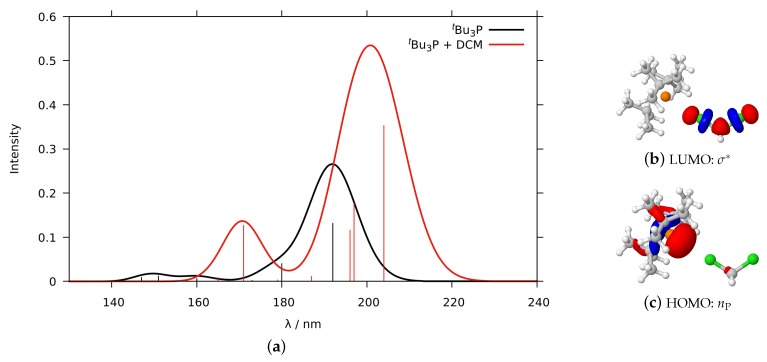
Calculated absorption spectra of ${}^{t}$Bu${}_{3}$P in DCM (black: continuum solvent model, red: ${}^{t}$Bu${}_{3}$P-DCM adduct surrounded by a continuum solvent model of DCM) and frontier molecular orbitals of the ${}^{t}$Bu${}_{3}$P-DCM monoadduct.

The first absorption band of the isolated ${}^{t}$Bu${}_{3}$P, ${}^{n}$Bu${}_{3}$P, and (MeO)${}_{3}$P molecules peak at wavelengths shorter than 200 nm (Figure S4). Only their tails can be seen in the experimental observation window. They involve the respective lone-pair orbital on phosphorus, $n_{P}$, and a pair of nearly degenerate $\sigma^{*}$ orbitals of the trialkylphosphine. In dichloromethane (DCM) solution, phophine–DCM adducts can be formed, with a marked impact on the photophysics (Figure 3a) [47,48,49]. The first excited singlet state of the adduct is an optically bright charge transfer (CT) state originating from the excitation of an $n_{P}$ electron on the electron-rich phosphine to a $\sigma^{*}$ orbital on DCM (Figure 3). This CT excitation is somewhat red-shifted with regard to the local phosphine excitations ($\lambda_{max}=205$ nm in ${}^{t}$Bu${}_{3}$P–DCM compared to $\lambda_{max}=192$ nm in ${}^{t}$Bu${}_{3}$P) but much stronger (oscillator strength f=0.353 in ${}^{t}$Bu${}_{3}$P–DCM compared to f=0.132 in ${}^{t}$Bu${}_{3}$P). The adduct complex is therefore expected to dominate the residual intensity of the ${}^{t}$Bu${}_{3}$P absorption in the low-energy regime. This assumption is supported by the experimental observation of a lower absorbance of ${}^{t}$Bu${}_{3}$P in non-coordinating solvents such as pentane in the wave length region >250 nm (cf. Figure S5).

#### 2.1.3. Phosphine–Perfluorobutyl Iodide Adducts

${}^{t}$Bu${}_{3}$P, ${}^{n}$Bu${}_{3}$P, and (MeO)${}_{3}$P form perfluorobutyl iodide adducts with nearly linear C–I–P coordination in the electronic ground state. In line with the donor capabilities of these phosphines, the elongation of the I–C bond is most pronounced in the ${}^{t}$Bu${}_{3}$P–IC${}_{4}$F${}_{9}$ adduct and smallest in (MeO)${}_{3}$P–IC${}_{4}$F${}_{9}$. (For details, see Table 1.)

**Figure 4:**
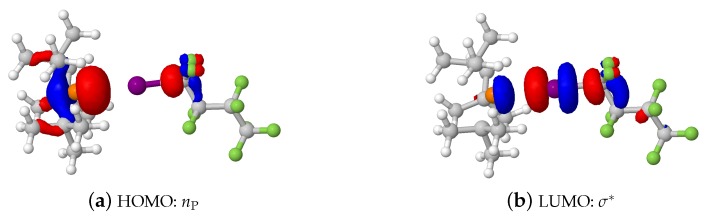
Frontier molecular orbitals of the ${}^{t}$Bu${}_{3}$P-IC${}_{4}$F${}_{9}$ adduct at the S${}_{0}$ geometry.

In all phosphine adducts, the HOMO is predominantly composed of the lone-pair orbital on phosphorus ($n_{P}$), whereas the LUMO is a $\sigma^{*}$-type orbital. MO plots are shown for ${}^{t}$Bu${}_{3}$P-IC${}_{4}$F${}_{9}$ in Figure 4, whereas those of the other adduct compounds may be found in Figures S6 and S7.

The direct T${}_{1}\leftarrow$S${}_{0}$ transitions of all phosphine adducts have negligible oscillator strengths in the Franck–Condon (FC) region. The strongly absorbing ${}^{1}\left(n_{P}\sigma^{*}\right)$ CT state forms the first excited singlet state of ${}^{t}$Bu${}_{3}$P-IC${}_{4}$F${}_{9}$. The triplet excitations from the two lone-pair orbitals on the iodine center ($n_{I}$) to the $\sigma^{*}$-orbital are close in energy but SOC between these states is not very pronounced. For this reason, the first S${}_{1}\leftarrow$S${}_{0}$ absorption maxima computed in the absence and presence of SOC are nearly identical (cf. Figure S8). A qualitatively similar energy scheme is obtained in the case of ${}^{n}$Bu${}_{3}$P-IC${}_{4}$F${}_{9}$ whose calculated absorption maximum is somewhat blue-shifted. In (MeO)${}_{3}$P-IC${}_{4}$F${}_{9}$, the much weaker ${}^{1}\left(n_{I}\sigma^{*}\right)$ transitions are energetically favored over the ${}^{1}\left(n_{P}\sigma^{*}\right)$ CT transition, but the states are close in energy. In line with the lower electron donor capabilities of (MeO)${}_{3}$P, the peak maximum is even more blue-shifted.

**Figure 5:**
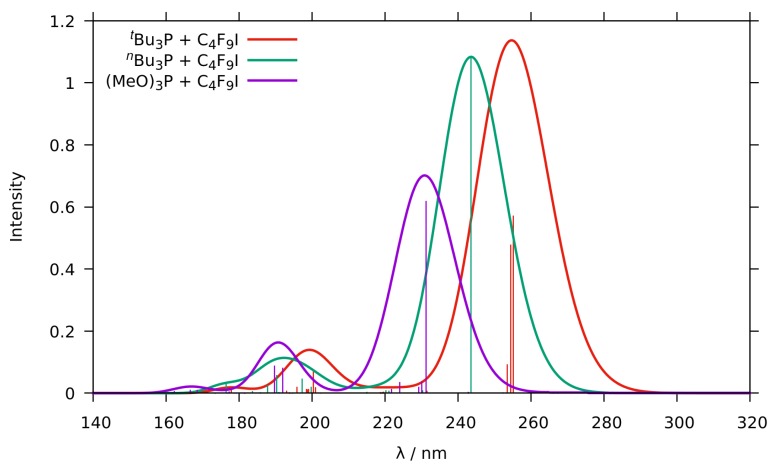
Calculated absorption spectra of the phosphine and phosphite adducts in DCM including spin–orbit coupling (SOC).

While the trends among the phosphine adducts are reproduced correctly, the computed absorption bands of the CT transitions (Figure 5) are red-shifted with respect to the corresponding experimental data (Figure 1) by about 20 nm. Note, however, that the transparency of the dichloromethane solvent quickly degrades for wavelengths shorter than 240 nm. To check the influence of the solvent on the absorption characteristics, the experiment was repeated for ${}^{t}$Bu${}_{3}$P-IC${}_{4}$F${}_{9}$ in pentane solution (transparency ≥90% up to 220 nm). And indeed, the first absorption band peaks at 255 nm in that solvent before the cut-off is reached (cf. Figures S2, S9 and S10).

Despite the good agreement between theory and experiment with regard to absorption maxima, quantum chemical calculations, performed at the respective ground-state geometries of the adducts, are not sufficient to explain the blue-light excitation of the compounds and their photochemistry. To this end, adiabatic excitation energies and minimum geometries of the lowest excited singlet and triplet states have to be known.

Similar to the basis set dependence of the C${}_{4}$F${}_{9}$I absorption, we observe a small red shift (4 nm) and a slight increase of the intensity of the first absorption band of the ${}^{t}$Bu${}_{3}$P-IC${}_{4}$F${}_{9}$ adduct when using a better atomic orbital basis set (cf. Figure S11). In view of the small changes and the markedly higher computational cost, we refrain from carrying out the elaborate excited-state geometry optimizations using the TZVP basis sets.

### 2.2. Relaxed Excited-State Geometries of the Phosphine–Perfluorobutyl Iodide Adducts

#### 2.2.1. The First Excited Triplet State

**Figure 6:**
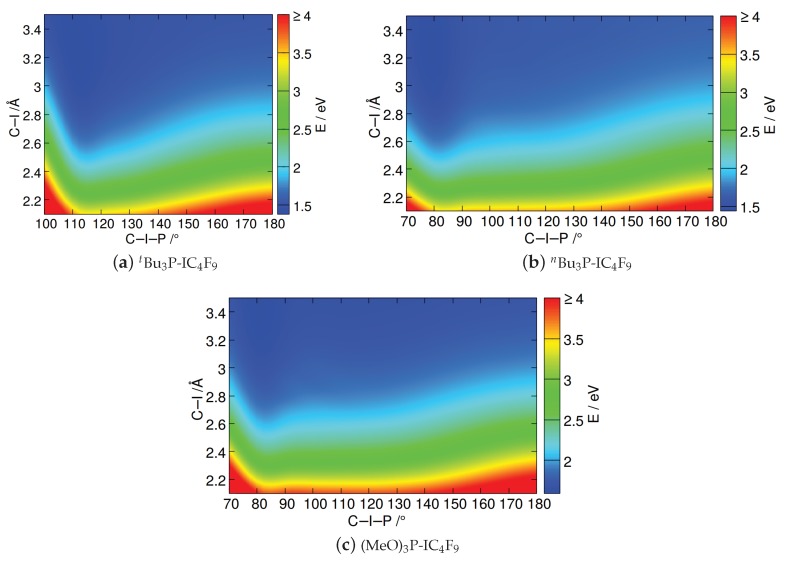
Heatmaps of the T${}_{1}$ potential energy surfaces of the phosphine and phosphite adducts as functions of the C–I bond length and the C–I–P angle. All other internal coordinates were kept fixed to the minimum geometry parameters of the respective T${}_{1}$ state. The energy scales on the right refer to DFT and multireference configuration interaction (DFT/MRCI) energies relative to the ground-state minimum. Note the different scales of the *x*-axes.

The T${}_{1}$ states form very shallow basins in the I–C dissociative region of the respective potential energy surfaces (PESs) (cf. heatmaps in Figure 6a–c).

**Figure 7:**
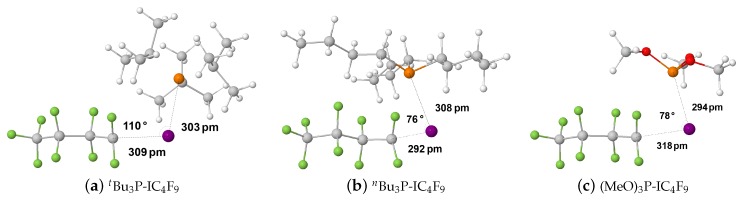
Nuclear arrangement of the phosphine–IC${}_{4}$F${}_{9}$ adducts at the optimized T${}_{1}$ minima.

Here, the biradicalic triplet is the lowest-energy state, and I–C bond cleavage can proceed nearly without any barrier. In addition to the markedly increased I–C bond length, we notice significant changes of the P–I–C bond angle with respect to the diamagnetic species (cf. Table 1). The P–I–C bond angle varies among the three adducts from approximately 110${}^{\circ }$ in ${}^{t}$Bu${}_{3}$P-IC${}_{4}$F${}_{9}$ for the phosphine with the largest steric demand to less than 80${}^{\circ }$ in ${}^{n}$Bu${}_{3}$P-IC${}_{4}$F${}_{9}$ and (MeO)${}_{3}$P-IC${}_{4}$F${}_{9}$ (Figure 7). The electronic structures of the T${}_{1}$ states (cf. Figure S12 for the singly occupied molecular orbitals, SOMOs) are in qualitative agreement with the picture of a perfluoroakyl radical attached to a negatively charged iodine and a phosphine radical cation (F${}_{9}$C${}_{4}^{•}\;\cdots {\;}^{⊖}$I${}^{•⊕}$PR${}_{3}$).

Adiabatically, the T${}_{1}$ minima are located between 1.42 and 1.62 eV (Table 2) above the corresponding S${}_{0}$ minima—too high in energy to be reached by thermal activation. Direct light activation is doomed to fail as well because the singlet–triplet mixing is too low in this CT state for making direct T${}_{1}\leftarrow$S${}_{0}$ absorption feasible (vide infra). The most probable activation pathway of the triplet channel is photoexcitation of the S${}_{1}$ state followed by ISC to the T${}_{1}$ PES.

#### 2.2.2. The First Excited Singlet State

**Figure 8:**
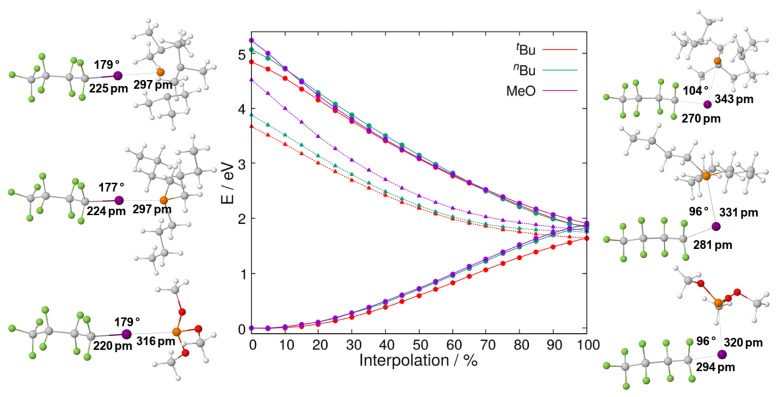
Energy profiles of the ground and first excited singlet states (solid lines) and the first excited triplet states (dotted lines) of the phosphine–IC${}_{4}$F${}_{9}$ adducts along linearly interpolated paths connecting the S${}_{0}$ minimum (left, 0%) and the optimized S${}_{1}$ geometry (right, 100%).

Unfortunately, minima on the S${}_{1}$ PESs of the phosphine-IC${}_{4}$F${}_{9}$ adducts could not be determined using the same computational protocol as for the T${}_{1}$ states. Upon relaxation of the S${}_{1}$ geometry at the time-dependent density functional theory (TDDFT)-Tamm–Dancoff approximation (TDA) level, the S${}_{1}$ and S${}_{0}$ states of all adducts undergo conical intersections where the calculation stops. Subsequent multireference configuration interaction (MRCI) single-point calculations revealed substantial double excitation contributions to the S${}_{o}$ and S${}_{1}$ wave functions at these geometries. We therefore preformed minimum searches based on numerical MRCI gradients to locate the lowest points on the S${}_{1}$ PESs. The nuclear arrangements at these minima are displayed in Figure 8 together with the course of the S${}_{0}$ and S${}_{1}$ potential energies along linearly interpolated paths connecting the singlet ground and excited state minima.

**Figure 9:**
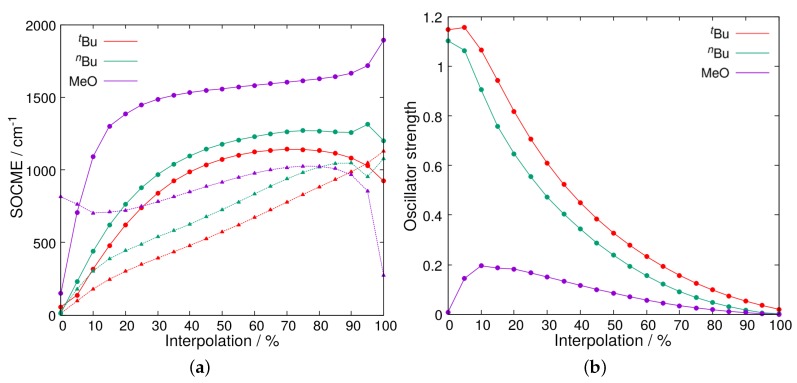
(**a**) Spin–orbit coupling matrix elements $\left|\langle T_{1}|\;{\hat{H}}_{so}\;|S_{0}\rangle \right|$ (dotted lines) and $\left|\langle T_{1}|\;{\hat{H}}_{so}\;|S_{1}\rangle \right|$ (solid lines) and (**b**) oscillator strengths of the S${}_{1}\leftarrow$S${}_{0}$ transitions along linearly interpolated paths connecting the S${}_{0}$ minimum (0%) and the optimized S${}_{1}$ geometry (100%).

While the S${}_{0}$ and S${}_{1}$ potentials of ${}^{t}$Bu${}_{3}$P-IC${}_{4}$F${}_{9}$ still exhibit a small energy gap of about 0.20 eV at the S${}_{1}$ minimum, S${}_{0}$ and S${}_{1}$ are practically degenerate at the relaxed S${}_{1}$ geometries of ${}^{n}$Bu${}_{3}$P-IC${}_{4}$F${}_{9}$ and (MeO)${}_{3}$P-IC${}_{4}$F${}_{9}$ and underdo conical intersections. In any case, the S${}_{0}$ and S${}_{1}$ wave functions are strongly mixed in the neighborhood, and non-adiabatic coupling is large. On the one hand, we therefore expect non-radiative deactivation by internal conversion (IC) to the electronic ground state to be fast in all phosphine adducts. On the other hand, the T${}_{1}$-dominated states lie energetically close as well ($\Delta E_{S_{1}-T_{1}}\leq 0.20$ eV) and their mutual spin–orbit couplings (Figure 9a) should suffice to transfer substantial population from S${}_{1}$ to T${}_{1}$ by ISC. Whether the photochemically active triplet channels can be reached depends on subtle differences between the competing IC and ISC dynamics.

The entries in Table 2 show that the origins of the S${}_{1}\leftarrow$S${}_{0}$ transitions of all phosphine adducts can, in principle, be reached by irradiation with the blue LED ($\lambda_{max}=461$ nm) used in the experiment. Despite the higher vertical excitation energy of (MeO)${}_{3}$P–IC${}_{4}$F${}_{9}$ in the FC region compared to ${}^{t}$Bu${}_{3}$P–IC${}_{4}$F${}_{9}$, we find nearly equal adiabatic S${}_{1}$-S${}_{0}$ energies in all adduct complexes. Moreover, while the computed oscillator strength of the S${}_{1}\leftarrow$S${}_{0}$ absorption of the ${}^{n}$Bu${}_{3}$P-IC${}_{4}$F${}_{9}$ adduct is somewhat smaller than in the ${}^{t}$Bu${}_{3}$P–IC${}_{4}$F${}_{9}$ adduct (Figure 9b), it is substantially larger than the corresponding quantity in the (MeO)${}_{3}$P–IC${}_{4}$F${}_{9}$ adduct. One may therefore wonder why the experimentally observed absorption intensity of the ${}^{n}$Bu${}_{3}$P-IC${}_{4}$F${}_{9}$ adduct is so much weaker than that of the two other compounds in the blue-light regime.

**Figure 10:**
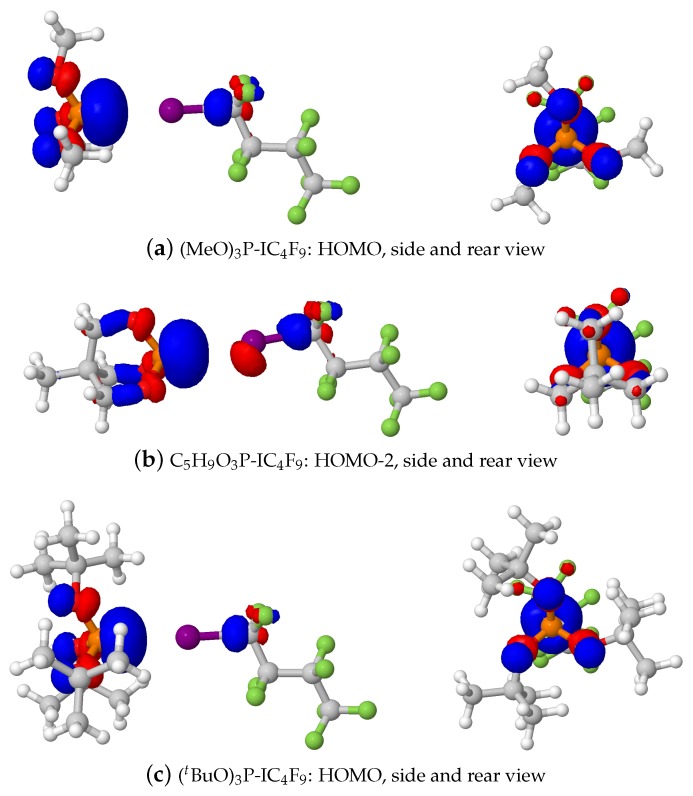
Frontier molecular orbitals of the phosphite–IC${}_{4}$F${}_{9}$ adducts at the S${}_{0}$ geometry.

On a related note, the high efficiency of (MeO)${}_{3}$P as catalyst in the iodoperfluoroalkylation contrasts with both the smaller maximal absorption and its weaker Lewis basicity. Moreover, it seems to be correlated with the broad tailing of the absorption into the visible light region. The HOMO of the (MeO)${}_{3}$P–IC${}_{4}$F${}_{9}$ adduct shows a substantial participation of the oxygen lone pairs (Figure 10a). This suggests a strong influence of the conformational flexibility of the alkoxide substituents upon the HOMO energy. This should lead to absorption band broadening due to a large ensemble of different conformers. Since a complete analysis of the conformational space and its corresponding excited states of F(CF${}_{2}$)${}_{n}$-I⋯P(OMe)${}_{3}$ was out of reach, we aimed at the spectroscopic and computational evaluation of different, conformationally locked phosphites, such as the “caged” phosphite 4-methyl-2,6,7-trioxa-1-phosphabicyclo[2.2.2]octane and tri(*tert*-butyl)phosphite (${}^{t}$BuO)${}_{3}$P [50,51,52]. In the case of the caged phosphite, the oxygen lone pairs point in the same direction as the phosphorus lone pair. Accordingly, substantial participation was not found in the HOMO but only in the HOMO-2 orbital (Figure 10b). In contrast, the highly rigid (${}^{t}$BuO)${}_{3}$P with the oxygen lone pairs pointing backwards shows their strong participation in the HOMO of the adduct with (I-P-O-C) dihedral angles of 62–64${}^{\circ }$ (Figure 10c).

**Figure 11:**
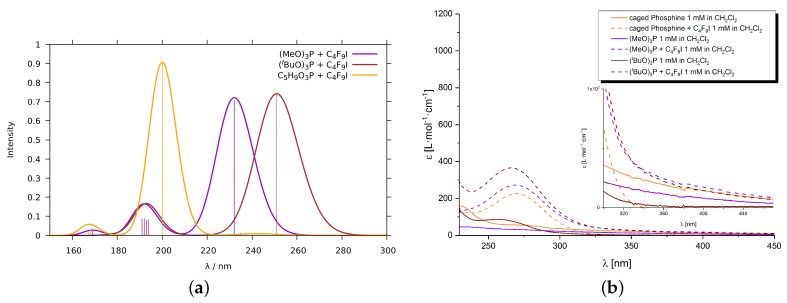
(**a**) Calculated and (**b**) measured absorption spectra of the three phosphite–IC${}_{4}$F${}_{9}$ adducts. In addition, experimental absorption spectra of the pure phosphites in DCM solution are shown.

The corresponding calculated absorption spectra (Figure 11a) for the three phosphite adducts show a substantial difference in excitation energies supporting this assumption further. These findings correlate with the experimental, spectroscopic analysis of the corresponding perfluoroalkyl iodide–phosphite complexes (Figure 11b). Complex formation in solution is less pronounced than in the case of phosphines. Therefore, absorption in the region of 240–320 nm is dominated by free perfluoroalkyl iodide. The caged phosphite shows very small broadening in absorption and was also completely inactive as catalysts; (${}^{t}$BuO)${}_{3}$P showed increased absorption and substantial tailing into the visible light region. It should be noted, however, that tailing was even more pronounced for (MeO)${}_{3}$P and a stronger absorption above 440 nm correlates with higher activity as catalyst.

## 3. Discussion

To understand the different photochemical behaviors of the adducts, we note that the probability $W_{\mathrm{rad}}^{\mathrm{FC}}\left(f-i\right)$ of a radiative transition from an initial electronic state *i* to a final electronic state *f*,

(1) $$W_{\mathrm{rad}}^{\mathrm{FC}}\left(f-i\right)=\frac{4e^{2}}{3c^{3}\hbar^{4}}\sum_{b}\sum_{a}{\left(E_{f,b}-E_{i,a}\right)}^{3}{\left|\langle \Psi_{f}|\hat{\mu }|\Psi_{i}\rangle \right|}^{2}{\left|\langle v_{f,b}|v_{i,a}\rangle \right|}^{2},$$

does not depend solely on the electric transition dipole moment $\left|\langle \Psi_{f}|\hat{\mu }|\Psi_{i}\rangle \right|$ and the energy difference ($E_{f}-E_{i}$) of the two states. Through the Franck–Condon factors ${\left|\langle v_{f,b}|v_{i,a}\rangle \right|}^{2}$, i.e., the squared overlaps of the vibrational wave functions, it depends on the coordinate displacements of the two potential minima as well. In Equation (1), *e* denotes the charge of the electron, *c* the velocity of light, and *ℏ* the Planck constant *h* divided by 2π.

Inspection of the molecular structures in Figure 8 reveals that the S${}_{1}$ minimum of the ${}^{t}$Bu${}_{3}$P-IC${}_{4}$F${}_{9}$ adduct lies geometrically somewhat closer to the corresponding ground-state equilibrium than the ${}^{n}$Bu${}_{3}$P-IC${}_{4}$F${}_{9}$ and (MeO)${}_{3}$P-IC${}_{4}$F${}_{9}$ adducts. The closer geometrical distance between the S${}_{0}$ and S${}_{1}$ minima is also reflected in the slightly shallower slope of the ground-state energy profile in Figure 8. In particular, the small overlap of the ground- and excited-state vibrational wave functions in the C–I–P angle bending coordinate of ${}^{n}$Bu${}_{3}$P-IC${}_{4}$F${}_{9}$ hampers the transition from the nearly linear ground-state equilibrium structure to the bent structure in the S${}_{1}$ potential well. This overlap is not large in ${}^{t}$Bu${}_{3}$P-IC${}_{4}$F${}_{9}$ either, but it appears sufficient to explain the stronger residual absorption of the S${}_{1}$ state in the blue wavelength region (cf. Figure 1). We note further that the oscillator strength of the S${}_{1}\leftarrow$S${}_{0}$ transition is the largest one among the three complexes (Table 2).

In all three cases, the S${}_{1}$ minimum lies geometrically in the exit channel of the T${}_{1}$ state toward C–I dissociation. The mutual SOC of the S${}_{1}$ and T${}_{1}$ states in this region implies that S${}_{1}⇝$T${}_{1}$ ISC is possible in competition with S${}_{1}⇝$S${}_{0}$ internal conversion back to the S${}_{0}$ minimum. Based on these data, we propose the following mechanism for the blue-light-initiated phosphine-catalyzed homolytic C–I cleavage of perfluorobutane iodide:

(2) $$\begin{array}{ccc} {}^{1}\;\left(R_{3}P+{\mathrm{IC}}_{4}F_{9}\right) & \to & \;{\;}^{1}\;\left(R_{3}P-{\mathrm{IC}}_{4}F_{9}\right)\overset{\begin{array}{c} \mathrm{blue}\;\mathrm{LED} \end{array}}{⇌}\;{\;}^{1}\;{\left(R_{3}P-{\mathrm{IC}}_{4}F_{9}\right)}^{*}\\ & \overset{\mathrm{ISC}}{⇝} & \;{\;}^{3}\;{\left(R_{3}P^{⊕•}-I^{⊖}\cdots C_{4}F_{9}\right)}^{*}\to R_{3}{\mathrm{PI}}^{\rceil •}+{\;}^{•}C_{4}F_{9}. \end{array}$$

While the different behavior of the phosphines ${}^{t}$Bu${}_{3}$P and ${}^{n}$Bu${}_{3}$P can be correlated primarily by their absorption profile, the surprisingly high reactivity of (some) phosphites may be the result of oxygen lone-pair participation and concomitant broadening of absorption. The strong tailing in the case of (MeO)${}_{3}$P and the associated relatively high absorption above 440 nm may be explained by its high conformational flexibility. Due to rotation about the P–O bond, energetically disfavored conformers are accessible in small fractions that show optimal orbital alignment for excitation. In the case of the conformationally locked (${}^{t}$BuO)${}_{3}$P, the oxygen lone pairs are already in a suitable position but the associated energy barriers are higher and the optimal geometry for excitation may be out of reach.

## 4. Materials and Methods

### 4.1. Computational Methods

Closed-shell ground-state geometries were optimized with resricted Kohn–Sham (KS) density functional theory (DFT) using Turbomole [53,54,55] in conjunction with the B3-LYP functional [56,57], Grimme D3-BJ dispersion corrections [58,59] and the continuum solvent model COSMO [60,61] for mimicking a DCM solvent environment. Unless stated otherwise, the def2-SVP atomic orbital basis sets [62] from the Turbomole library were utilized for all atoms, save for iodine, which was represented by a relativistic small-core effective core potential and the corresponding def2-TZVPD basis [63]. To check the sensitivity of the results with respect to the choice of atomic orbital basis set, additional test calculations on the absorption properties of C${}_{4}$F${}_{9}$I and its ${}^{t}$Bu${}_{3}$P adduct were performed employing the larger def2-TZVP basis sets for H, C, F, and P [64,65], while keeping the iodine basis set unmodified. Triplet geometry optimizations were performed with the corresponding time-dependent DFT (TDDFT) [66] module using the Tamm–Dancoff approximation [67] (TDA) to TDDFT. In (MeO)${}_{3}$P-IC${}_{4}$F${}_{9}$, where TDDFT-TDA did not converge, we employed unrestricted KS DFT instead for the geometry optimization of the triplet state. Numerical second derivatives for vibrational analyses were computed with SNF [68].

Minimum searches at the TDDFT level were not successful for the excited singlet states of the adducts. In these cases, we employed a computational protocol based on numerical DFT/MRCI gradients [69]. The combined DFT and multireference configuration interaction (DFT/MRCI) [70,71] approach was used in conjunction with the semiempirical R2018 Hamiltonian [72] and the standard configuration selection threshold of 1.0 E${}_{h}$ to determine spin–orbit free excitation energies and oscillator strengths. The KS orbitals and orbital energies for the DFT/MRCI calculations were optimized utilizing the BH-LYP [57,73] density functional, empirical dispersion corrections [58,59], and an implicit DCM solvent environment [60,61]. Auxiliary basis sets for the resolution-of-the-identity approximation of the two-electron integrals [70,74] were taken from the Turbomole library [55], i.e., we chose the def2-TZVPD auxiliary basis set [75] on iodine and the def2-SVP sets [64] on all other atoms.

Interstate spin–orbit coupling matrix elements (SOCMEs) for determining the probability of ISC were computed by the Spock program [76,77,78] using a spin–orbit effective core potential for iodine [63] and an effective one-center mean-field approximation to the Breit–Pauli SOC operator [79,80] for the lighter elements. For efficiency reasons, vertical excitation energies and oscillator strengths of multiplicity-mixed wave functions were determined by quasi-degenerate perturbation theory (QDPT) in the basis of DFT/MRCI wave functions. Adiabatic excitation energies including SOC were obtained by means of multireference spin–orbit configuration interaction (MRSOCI) [81] calculations.

### 4.2. Experimental Procedures

All preparations involving air- and moisture-sensitive compounds were carried out inside a glove box (*Vacuum Atmospheres* model OMNI-LAB) under N${}_{2}$ atmosphere (*Air Liquide ALPHAGAZ*${}^{\mathrm{TM}}$ 5.0). Glassware was dried for 2 h at 120 ${}^{\circ }$C and cooled down in vacuo.

Nonafluoro-1-iodobutane was purchased from TCI and was filtered through a column packed with aluminum oxide 90 basic 0.063–0.200 mm (activity stage I) and an activated molecular sieve (4 Å) under N${}_{2}$ atmosphere. The clear and colorless liquid was stored in amber glass vials under N${}_{2}$ atmosphere. Tri-tert-butylphosphine was purchased from Sigma Aldrich. 4-Methyl-2,6,7-trioxa-1- phosphabicyclo[2.2.2]octane [82] and tri(*tert*-butyl)phosphite [50,83] were prepared according to the literature.

Pentane and dichloromethane were dried with the solvent purification system MP-SPS 800 from M. Braun and degassed with freeze-pump-thaw. UV-vis measurements were performed on a Perkin Elmer Lamda 2 UV-vis spectrometer in Hellma cuvettes (10 × 10 mm, Suprasil quartz glass).

## 5. Conclusions

The results of the present computational chemistry study support the mechanism of a phosphine-assisted light-induced homolytic C–I bond cleavage, proposed earlier by some of us [40] on the basis of experimental observations. In addition, they provide an explanation for the fact that ${}^{t}$Bu${}_{3}$P is a much better photocatalyst in the blue-light regime than ${}^{n}$Bu${}_{3}$P. While the origin of the S${}_{1}\leftarrow$S${}_{0}$ absorption band should be energetically accessible to blue-light radiation in both butylphosphine–IC${}_{4}$F${}_{9}$ adducts, the larger coordinate displacements of the ground and excited singlet potential energy wells in ${}^{n}$Bu${}_{3}$P-IC${}_{4}$F${}_{9}$ reduce the overlaps of the vibrational wave functions and thus the absorption probability compared to ${}^{t}$Bu${}_{3}$P-IC${}_{4}$F${}_{9}$. Due to the presence of iodine and its involvement in the electronic transitions, spin–orbit coupling is strong enough to enable intersystem crossing and to facilitate the population of the biradicalic triplet state, which is the photochemically active state.

The fact that both ${}^{t}$Bu${}_{3}$P and (MeO)${}_{3}$P are active catalysts for the iodoperfluoroalkylation can be rationalized by two different factors. In the case of the bulky phosphine as strong Lewis base donor, the overall increase in absorption results in a tailing into the visible light region sufficient for radical generation. In the case of the electron-deficient, conformationally flexible phosphite, the overall increase of absorption is only small, but efficient oxygen lone-pair participation in the HOMO, found in a small fraction of conformers, may be the reason for the observed broad tailing into the longer wavelength region and concomitant catalytic activity.

## Figures and Tables

**Table 1:** Selected geometry parameters of perfluorobutyl iodide and its phosphine and phosphite adducts in the electronic ground state (S${}_{0}$), the first excited triplet (T${}_{1}$), and singlet (S${}_{1}$) state.

Compound	P–I Bond Length/pm				I–C Bond Length/pm				P–I–C Bond Angle/${}^{\circ }$		
	S${}_{0}$	T${}_{1}$	S${}_{1}$		S${}_{0}$	T${}_{1}$	S${}_{1}$		S${}_{0}$	T${}_{1}$	S${}_{1}$
C${}_{4}$F${}_{9}$I					216						
${}^{t}$Bu${}_{3}$P-IC${}_{4}$F${}_{9}$	297	303	343		225	309	270		179	110	104
${}^{n}$Bu${}_{3}$P-IC${}_{4}$F${}_{9}$	297	308	331 ${}^{a}$		224	292	281 ${}^{a}$		177	76	96 ${}^{a}$
(MeO)${}_{3}$P-IC${}_{4}$F${}_{9}$	316	294	320 ${}^{a}$		220	318	294 ${}^{a}$		179	78	96 ${}^{a}$

${}^{a}$ S${}_{1}$/S${}_{0}$ conical intersection.

**Table 2:** Vertical and adiabatic excitation energies ($\Delta E_{vert}$ and $\Delta E_{adia}$/eV) and corresponding transition wavelengths ($\lambda_{vert}$ and $\lambda_{adia}$/nm) including SOC at the DFT/multireference spin–orbit configuration interaction (MRSOCI) level of theory. Triplet energies have been averaged over the three states with largest T${}_{1}$ contributions.

Compound	T${}_{1}$					S${}_{1}$			
	$\Delta E_{\mathrm{vert}}$	$\lambda_{\mathrm{vert}}$	$\Delta E_{\mathrm{adia}}$	$\lambda_{\mathrm{adia}}$		$\Delta E_{\mathrm{vert}}$	$\lambda_{\mathrm{vert}}$	$\Delta E_{\mathrm{adia}}$	$\lambda_{\mathrm{adia}}$
${}^{t}$Bu${}_{3}$P-IC${}_{4}$F${}_{9}$	3.70	335	1.42	870		4.84	256	1.70	729
${}^{n}$Bu${}_{3}$P-IC${}_{4}$F${}_{9}$	3.91	317	1.46	850		5.07	245	1.82 ${}^{a}$	681 ${}^{a}$
(MeO)${}_{3}$P-IC${}_{4}$F${}_{9}$	4.48	276	1.62	765		5.34	232	1.87 ${}^{a}$	663 ${}^{a}$

${}^{a}$ S${}_{1}$/S${}_{0}$ conical intersection.

## Supplementary Materials

The following are available online. Description of general experimental procedures, synthesis of phosphites, reactions, NMR spectra, UV-vis measurements and further computational details, Figure S1: Calculated absorption spectra of C${}_{4}$F${}_{9}$I in CH${}_{2}$Cl${}_{2}$ with and without SOC, Figure S2: Experimental UV-vis spectra of C${}_{4}$F${}_{9}$I, ${}^{t}$Bu${}_{3}$P, and ${}^{t}$Bu${}_{3}$P + C${}_{4}$F${}_{9}$I in CH${}_{2}$Cl${}_{2}$, Figure S3: Atomic orbital basis set dependence of the calculated absorption spectrum of C${}_{4}$F${}_{9}$I (190–400 nm) in CH${}_{2}$Cl${}_{2}$, Figure S4: Computed absorption spectra of the phosphines (${}^{t}$Bu${}_{3}$P, ${}^{n}$Bu${}_{3}$P) and the phosphite (MeO)${}_{3}$P in CH${}_{2}$Cl${}_{2}$ with SOC, Figure S5: Comparison of the experimental UV-vis spectra of ${}^{t}$Bu${}_{3}$P in pentane and in CH${}_{2}$Cl${}_{2}$, Figure S6: Frontier molecular orbitals of the ${}^{n}$Bu${}_{3}$P-IC${}_{4}$F${}_{9}$ adduct, Figure S7: Frontier molecular orbitals of the (MeO)${}_{3}$P-IC${}_{4}$F${}_{9}$ adduct, Figure S8: Calculated absorption spectra of ${}^{t}$Bu${}_{3}$P–C${}_{4}$F${}_{9}$I in CH${}_{2}$Cl${}_{2}$ with and without SOC, Figure S9: Experimental UV-vis spectra of C${}_{4}$F${}_{9}$I, ${}^{t}$Bu${}_{3}$P, and ${}^{t}$Bu${}_{3}$P + C${}_{4}$F${}_{9}$I in pentane, Figure S10: Comparison of the experimental UV-vis spectra of ${}^{t}$Bu${}_{3}$P + C${}_{4}$F${}_{9}$I in pentane and in CH${}_{2}$Cl${}_{2}$, Figure S11: Atomic orbital basis set dependence of the calculated DFT/MRCI singlet absorption spectrum of the ${}^{t}$Bu${}_{3}$P–C${}_{4}$F${}_{9}$I adduct complex, Figure S12: Singly occupied MOs (SOMOs) of the phosphine and phosphite adducts in the relaxed T${}_{1}$ state, Figures S13–S18: Solvent influence on the measured absorption spectra, Figures S19–S23: Impact of spin–orbit coupling on the calculated spectra, Figures S24–S39: Minimum nuclear arrangements with selected geometry parameters.

## Abbreviations

The following abbreviations are used in this manuscript:

ATRA	atom transfer radical addition
CT	charge transfer
DCM	dichloromethane
DFT	density functional theory
EDA	electron donor–acceptor
HOMO	highest occupied molecular orbital
IC	internal conversion
ISC	intersystem crossing
KS	Kohn–Sham
LED	light-emitting diode
LUMO	lowest unoccupied molecular orbital
MRCI	multi-reference configuration interaction
MRSOCI	multi-reference spin–orbit configuration interaction
PES	potential energy surface
QDPT	quasi-degenerate perturbation theory
SOC	spin–orbit coupling
SOCME	spin–orbit coupling matrix element
SOMO	singly occupied molecular orbital
TDDFT	time-dependent density functional theory
TDA	Tamm–Dancoff approximation
