# Supplementary material for: Visible Light-Induced Homolytic Cleavage of Perfluoroalkyl Iodides Mediated by Phosphines
Source: Molecules. 2020 Apr 1;25(7):1606. doi: 10.3390/molecules25071606 (PMC7181301; doi:10.3390/molecules25071606)
Supplement: Supplementary file 1 [file molecules-25-01606-s001.pdf]

Supplementary Materials: Visible Light-Induced Homolytic Cleavage of Perfluoroalkyl Iodides Mediated by Phosphines

Mario Bracker [1], Lucas Helmecke [2], Martin Kleinschmidt [1]*, Constantin Czekelius [2,]* and Christel M. Marian [1,]*

March 30, 2020

Contents

S1 General Experimental Procedures **2**

S2 Synthesis of phosphites **2**

S2.1 4-Methyl-2,6,7-trioxa-1-phosphabicyclo-[2,2,2]-octane (caged phosphite) 2

S2.2 Tri-*tert*-butyl phosphite 3

S3 Reactions **4**

S3.1 Reaction with 4-methyl-2,6,7-trioxa-1-phosphabicyclo-[2,2,2]-octane (caged phosphite) . 4

S3.2 Reaction with tri-*tert*-butylphosphite 4

S4 NMR Spectra **5**

S4.1 Caged phosphite 5

S4.2 $(^t\mathrm{BuO})_3\mathrm{P}$ 7

S5 UV-Vis Measurements **10**

S6 Further Computational Details **10**

S7 Absorption Spectra and Molecular Orbitals **11**

S7.1 Perfluorobutyl Iodide 11

S7.2 Phosphines and Phosphites 12

S7.3 Phosphine and Phosphite Adducts 13

S7.4 Solvent Influence on the Measured Absorption Spectra 16

S7.5 Impact of Spin–Orbit Coupling on the Calculated Spectra 20

S8 Minimum Nuclear Arrangements **22**

S8.1 DFT-Optimized Ground-State Geometries 22

S8.2 TDDFT/TDA-Optimized Conical Intersection Geometries 30

S8.3 TDDFT/TDA-Optimized Triplet Geometries 31

References **33**

S1. General Experimental Procedures

All preparations involving air- and moisture-sensitive compounds were carried out inside a glove box (*Vacuum Atmospheres* model OMNI-LAB) under N_2 atmosphere (*Air Liquide ALPHAGAZ*™ 5.0). Glassware was dried for 2 hours at 120 °C and cooled down in vacuo.

Nonafluoro-1-iodobutane was purchased from TCI and was filtered through a column packed with aluminum oxide 90 basic 0.063 - 0.200 mm (activity stage I) and activated molecular sieve (4 Å) under N_2 atmosphere. The clear and colorless liquid was stored in amber glass vials under N_2 atmosphere. Tri-*tert*-butylphosphine was purchased from Sigma Aldrich.

Pentane and dichloromethane were dried with the solvent purification system MP-SPS 800 from M.Braun and degassed with freeze-pump-thaw.

^{1}H-, ^{13}C, ^{31}P-spectra were recorded on *Bruker* Avance III 300 and 600. Chemical shifts are reported in parts per million (ppm) to the corresponding solvent. The order of citation in parentheses is a) multiplicity (s = singlet, d = doublet, m = multiplet), b) coupling constants, c) number of protons, and d) assignment. Coupling constants (J) were reported in Hertz (Hz). If not described differently, the NMR-spectra were measured at 298 K.

UV-VIS spectra were measured on a Perkin Elmer Lamda 2 UV-VIS spectrometer in Hellma cuvettes (10 x 10 mm, Suprasil quartz glass).

GC measurements were performed on a Shimadzu GC-2010 equipped with an auto injector AOC-20i (syringe code: 10R-S-0.63C). A ZB-Wax Plus column (30 $\times$ 0.25 mm $\times$ 0.25 μm) was used. As internal standard *n*-decane (Acros Organics, purity 99 + %, LOT:1283567) was added to the reaction solution. The used photoreactor is self-assembled and is described in literature. [1]

S2. Synthesis of phosphites

S2.1. 4-Methyl-2,6,7-trioxa-1-phosphabicyclo-[2,2,2]-octane (caged phosphite)

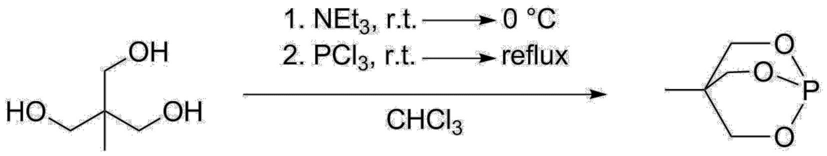

The synthesis of 4-methyl-2,6,7-trioxa-1-phosphabicyclo-[2,2,2]-octane was conducted similar to a literature known procedure. [2] In a 250 ml two-necked round-bottom flask with condenser tris(hydroxymethyl)ethane (7.21 g, 60.0 mmol, 1.0 equiv) and triethylamine (19.2 ml, 138 mmol, 2.3 equiv) were dissolved in $CHCl_3$ (70 ml). PCl_3 (5.2 ml, 59 mmol) in $CHCl_3$ (10 ml) was added dropwise at 0 °C to the cloudy reaction solution. After removing the ice bath the reaction solution was clear and was refluxed for 12 h. The clear reaction solution was extracted with desalinated water (3 $\times$ 50 ml), dried over anhydrous $MgSO_4$, filtered and the solvent was evaporated. The obtained colorless gel-like crystals were dissolved in CH_2Cl_2 (4 ml) and the solvent was evaporated again yielding colorless crystals.

yield (148.1 g mol^{-1})	4.70 g (31.7 mmol, 53%)

^{1}H-NMR (600 MHz, $CDCl_3$) δ [ppm] 3.94 (d, J = 1.9 Hz, CH_2, 6H), 0.73 (s, CH_3, 3H)

^{13}C-NMR (75.5 MHz, $CDCl_3$) δ [ppm] 71.94 (s, C—CH_3), 32.13 (d, J = 22.5 Hz, CH_2), 16.82 (d, J = 5.5 Hz, CH_3)

^{31}P-NMR (243 MHz, $CDCl_3$) δ [ppm] 91.2

Mp: 91.2 – 96.9 °C

Analytic data are consistent with literature-known values. [2,3]

*S2.2. Tri-*tert*-butyl phosphite*

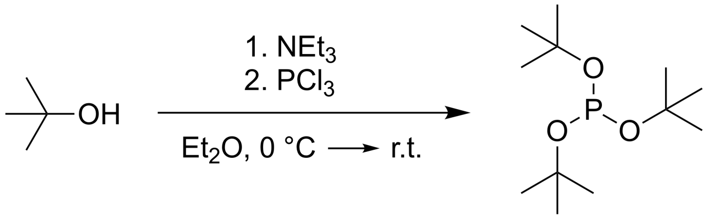

The synthesis of tri-*tert*-butyl phosphite was conducted similar to a literature known procedure. [4] Anhydrous diethyl ether was degassed with freeze-pump-thaw and each educt was degassed in Et_2O again before it was added. *Tert*-butyl alcohol (11.7 ml, 0.125 mol, 2.94 equiv) in Et_2O (25 ml) and triethylamine (17.3 ml, 0.125 mol, 2.94 equiv) in Et_2O (25 ml) were added together at 0 °C. PCl_3 (3.70 ml, 0.0425 mol) in Et_2O (12 ml) was added slowly via a dropping funnel, so that the reaction temperature maintained between 0 °C and 5 °C. After the addition was completed, Et_2O (30 ml) was added to the reaction solution and the reaction mixture was stirred 1 h at 0 °C and 16 h at r.t.. The reaction solution was separated via Schlenk filtration and the solvent was removed in vacuo. While the solvent was removed the round-bottom flask was cooled with an ice/water bath. A pale yellow oil was obtained, transferred into the glovebox and filtered through a syringe filter.

yield (250.3 g mol^{-1})	871.6 mg (3.48 mmol, 8%)

^{1}H-NMR (300 MHz, C_6D_6) δ [ppm] 1.39 (s, $(CH_3)_3C$).

^{13}C-NMR (75.5 MHz, C_6D_6) δ [ppm] 76.1 (s, $(CH_3)_3C$), 31.4 (s, $(CH_3)_3C$)

^{31}P-NMR (121 MHz, C_6D_6) δ [ppm] 151.1

^{1}H-NMR (300 MHz, $CDCl_3$) δ [ppm] 1.28 (s, $(CH_3)_3C$).

^{13}C-NMR (75.5 MHz, $CDCl_3$) δ [ppm] 75.8 (d, J = 6.0 Hz, $(CH_3)_3C$), 31.1 (d, J = 8.1 Hz, $(CH_3)_3C$)

^{31}P-NMR (121 MHz, $CDCl_3$) δ [ppm] 140.1

Analytic data are consistent with literature-known values. [4–6]

S3. Reactions

S3.1. Reaction with 4-methyl-2,6,7-trioxa-1-phosphabicyclo-[2,2,2]-octane (caged phosphite)

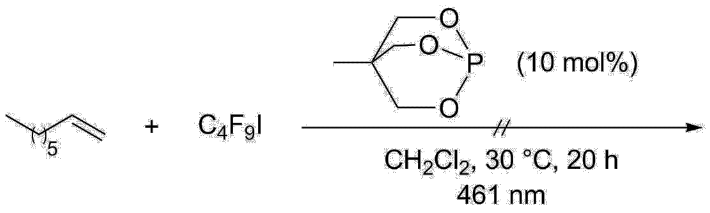

Caged phosphite (8.0 mg, 0.054 mmol, 10 mol%) was weighed into a 4 ml screw neck glass vial. Under a stream of nitrogen 1-octene (84 μl, 0.530 mmol) and CH_2Cl_2 (2 ml) were added. Under red light and the stream of nitrogen C_4F_9I (100 μl, 0.583 mmol, 1.10 equiv) was added, the vial was sealed with a septa screw cap and the reaction solution was irradiated (461 nm) for 20 h. After 1 h, 4 h and 20 h samples for a control by NMR spectroscopy were withdrawn under a stream of nitrogen and under red light. No conversion was observed.

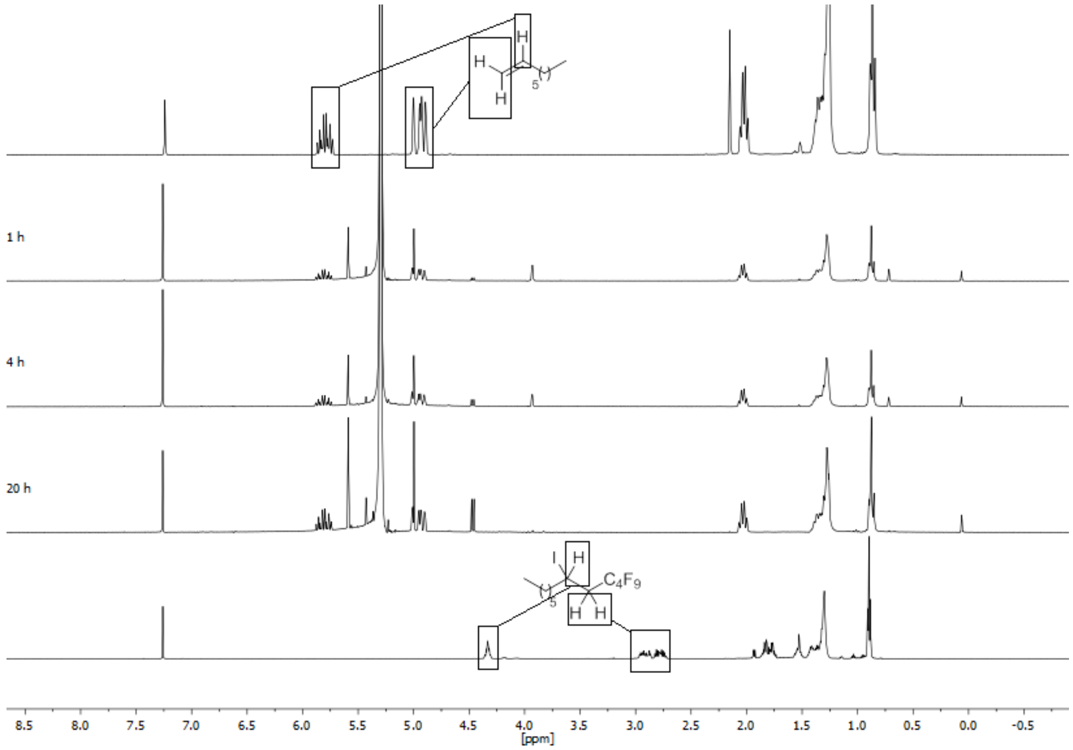

^{1}H-NMR-spectra (300 MHz, $CDCl_3$) of the reaction after 1 h, 4 h and 20 h in comparison with spectra of 1-octene (top) and the iodo perfluoroalkylation products (bottom).

*S3.2. Reaction with tri-*tert*-butylphosphite*

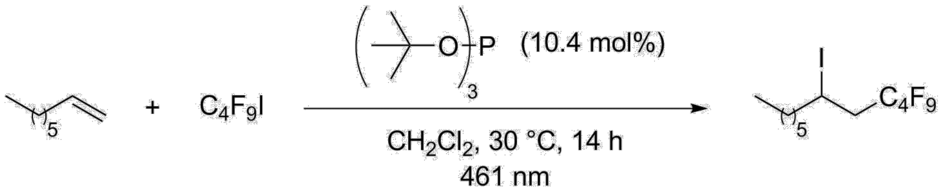

Inside the glovebox tri-*tert*-butylphoshite (14.4 mg, 0.0575 mmol, 10.4 mol%), *n*-decane (29.6 mg) and 1-octene (62.0 mg, 0.552 mmol) were weighed into a 4 ml screw neck glass vial. A Teflon stirring bar and CH_2Cl_2 (2 ml) were added. Under red light C_4F_9I (100 μl, 0.583 mmol, 1.05 equiv) was added, the vial was sealed with a septa screw cap and the reaction solution was irradiated (461 nm) for 14 h. After 1 h (conversion: 31%), 2 h (conversion: 51%) and 14 h (conversion: 56%) samples for a reaction control by GC were withdrawn under a stream of nitrogen. With a 1.0 ml syringe (Braun) flushed with N_2 0.10 ml of the reaction solution were withdrawn and diluted with 0.4 ml CH_2Cl_2 in a short amber thread vial. The vial was sealed with a black screw cap.

S4. NMR Spectra

S4.1. Caged phosphite

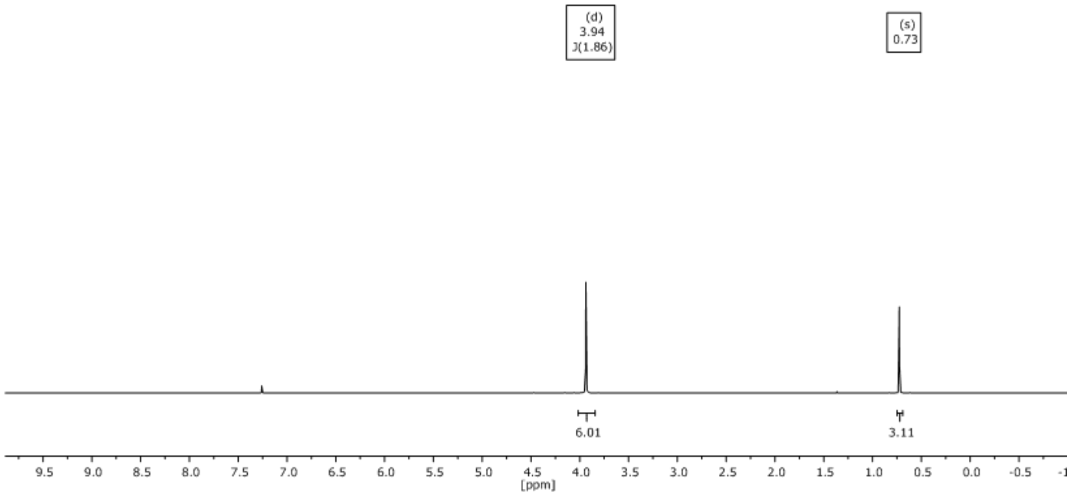

^{1}H-NMR-spectrum (600 MHz. $CDCl_3$)

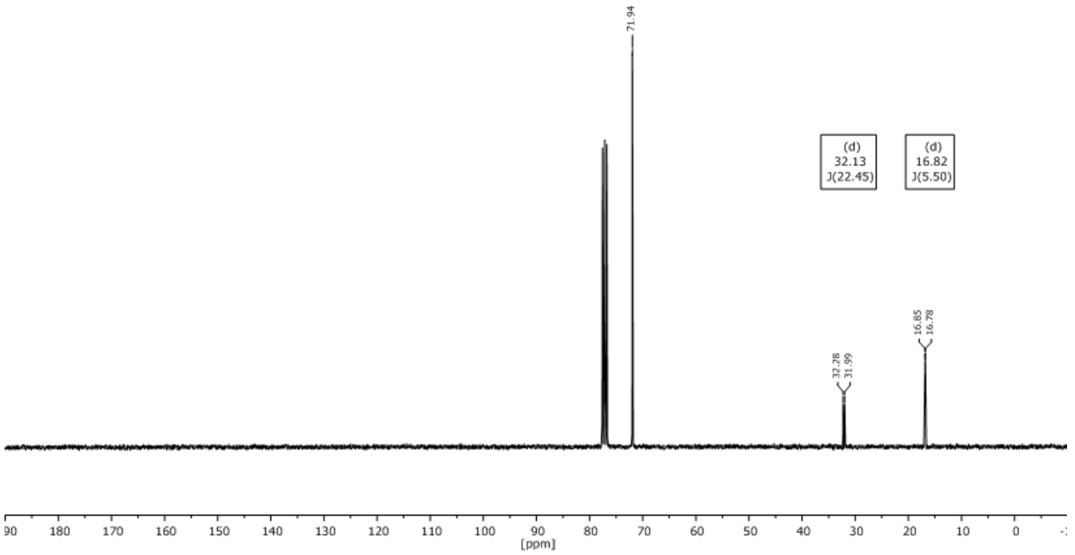

^{13}C-NMR-spectrum (75.5 MHz. $CDCl_3$)

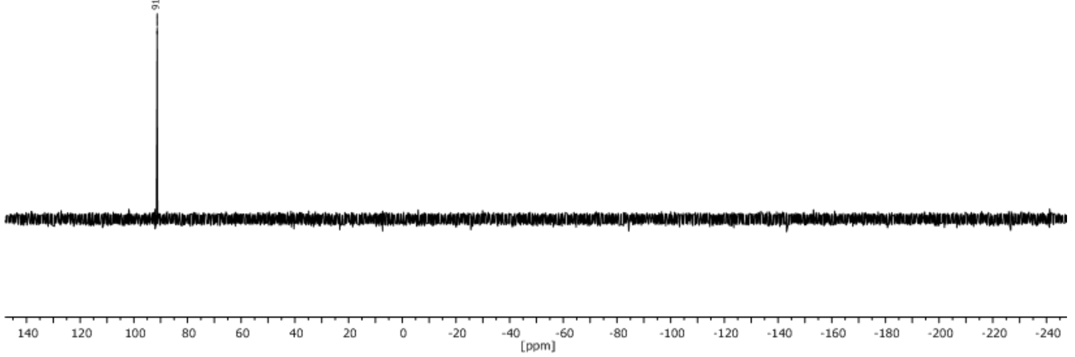

^{31}P-NMR-spectrum (243 MHz. $CDCl_3$)

S4.2. (^{t}BuO)$_3$P

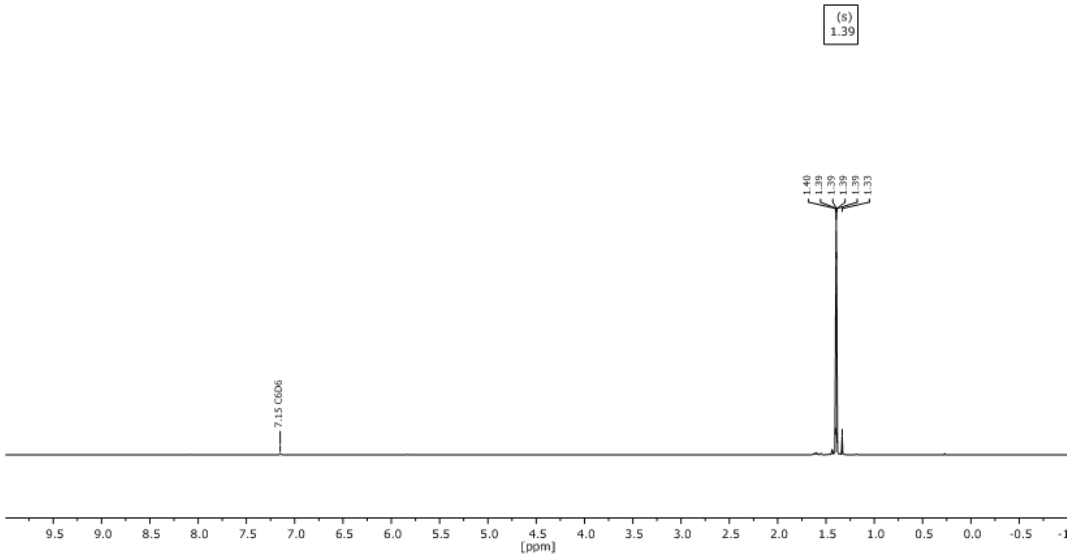

^{1}H-NMR-spectrum (300 MHz. C_6D_6)

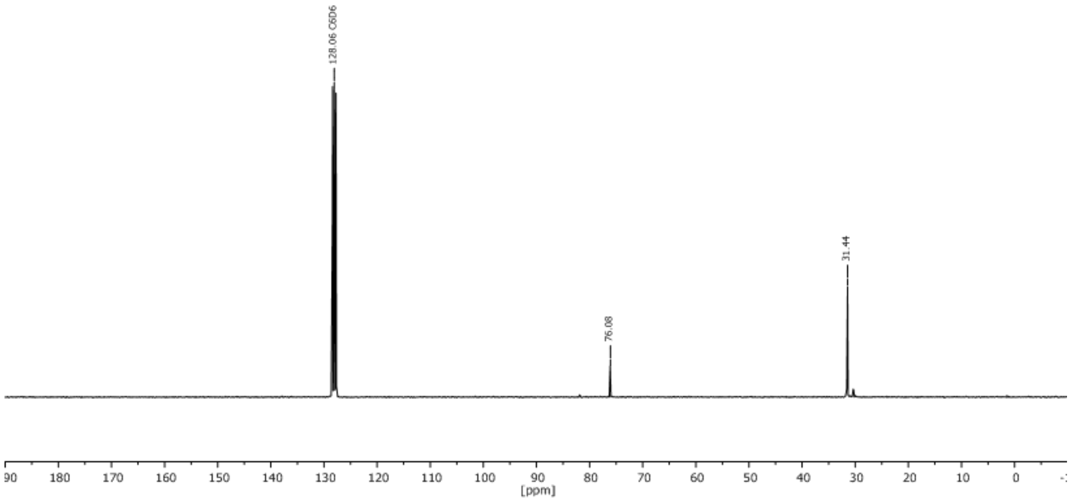

^{13}C-NMR-spectrum (75.5 MHz. C_6D_6)

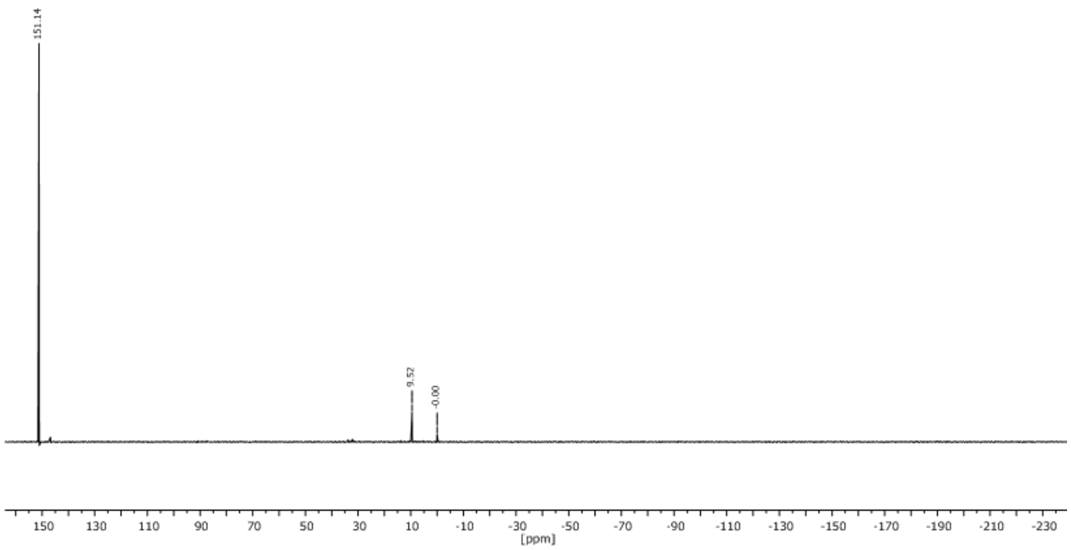

^{31}P-NMR-spectrum (121 MHz. $CDCl_3$)

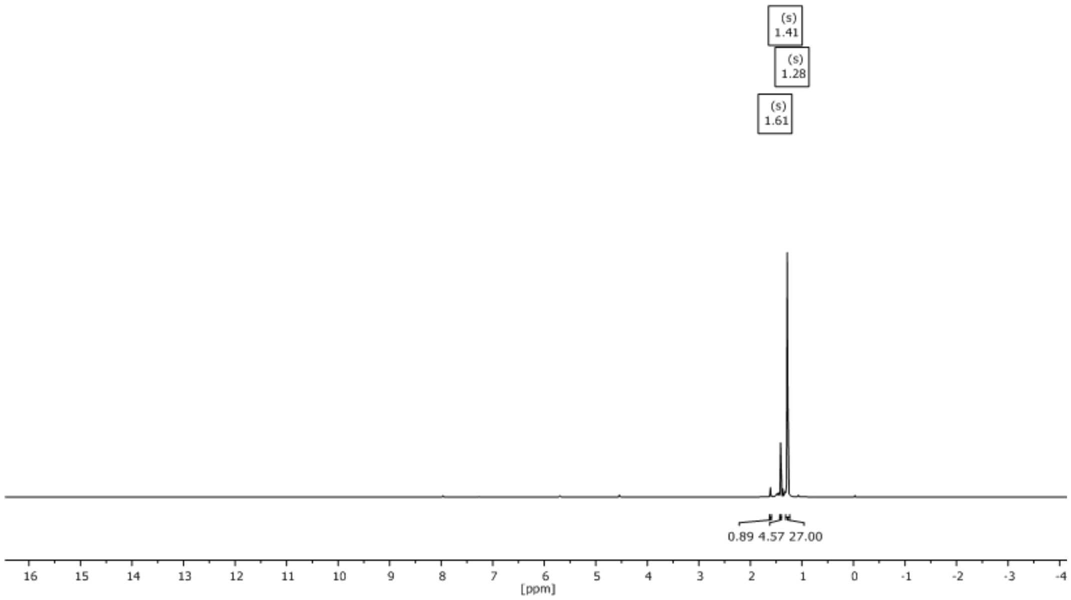

^{1}H-NMR-spectrum (300 MHz. $CDCl_3$)

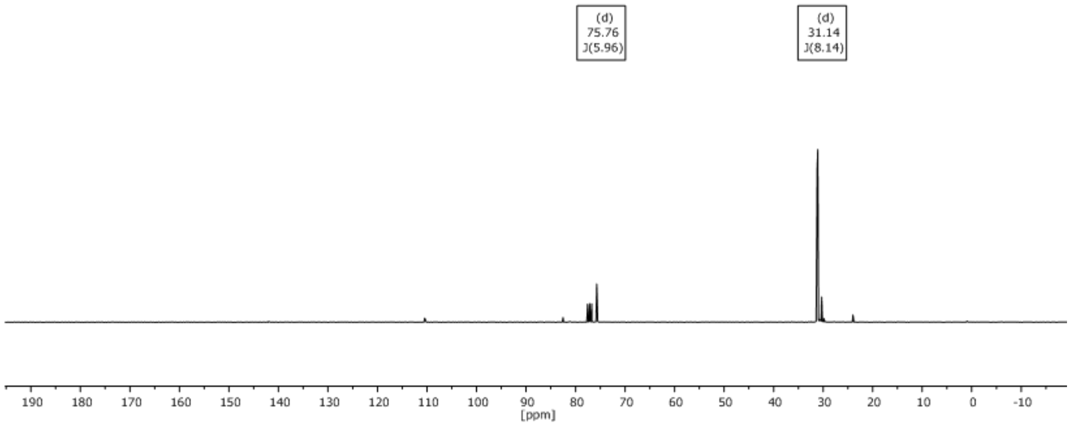

^{13}C-NMR-spectrum (75.5 MHz. $CDCl_3$)

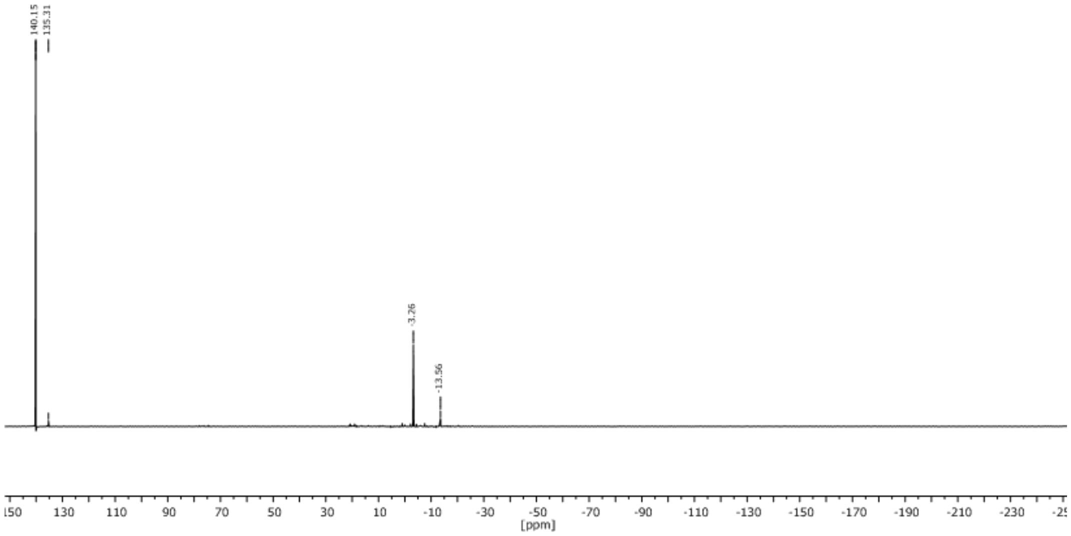

^{31}P-NMR-spectrum (121 MHz. $CDCl_3$)

S5. UV-Vis Measurements

- C_4F_9I (34.6 mg, 0.100 mmol) was dissolved in pentane or CH_2Cl_2 (10.0 ml) inside the glovebox in a volumetric flask. This solution was diluted in a volumetric flask (200 μL in 2.00 ml). The following concentration was present: [C_4F_9I] = 1.0 mM.
- ^{t}Bu$_3$P (20.2 mg, 0.100 mmol) was dissolved in pentane or CH_2Cl_2 (10.0 ml) in a volumetric flask inside the glovebox. This solution was diluted in a volumetric flask (200 μL in 2.00 ml). The following concentration was present: [^{t}Bu$_3$P] = 1 mM.
- ^{t}Bu$_3$P (20.2 mg, 0.100 mmol) and C_4F_9I (34.6 mg, 0.100 mmol) were dissolved in pentane or CH_2Cl_2 (10.0 ml) in a volumetric flask inside the glovebox. This solution was diluted in a volumetric flask (200 μL in 2.00 ml). The following concentration was present: [^{t}Bu$_3$P] = 1 mM, [C_4F_9I] = 1 mM.
- caged phosphine (14.8 mg, 0.100 mmol) was dissolved in CH_2Cl_2 (10.0 ml) in a volumetric flask. This solution was diluted in a volumetric flask (200 μL in 2.00 ml). The following concentration was present: [caged phosphine] = 1.0 mM.
- caged phosphine (14.8 mg, 0.100 mmol) and C_4F_9I (34.6 mg, 0.100 mmol) were dissolved in CH_2Cl_2 (10.0 ml) in a volumetric flask. This solution was diluted in a volumetric flask (200 μL in 2.00 ml). The following concentration was present: [caged phosphine] = 1 mM, [C_4F_9I] = 1 mM.
- (MeO)$_3$P (12.4 mg, 0.100 mmol) was dissolved in CH_2Cl_2 (10.0 ml) in a volumetric flask. This solution was diluted in a volumetric flask (200 μL in 2.00 ml). The following concentration was present: [(MeO)$_3$P] = 1.0 mM.
- (MeO)$_3$P (12.4 mg, 0.100 mmol) and C_4F_9I (34.6 mg, 0.100 mmol) were dissolved in CH_2Cl_2 (10.0 ml) in a volumetric flask. This solution was diluted in a volumetric flask (200 μL in 2.00 ml). The following concentration was present: [(MeO)$_3$P] = 1 mM, [C_4F_9I] = 1 mM.
- (^{t}BuO)$_3$P (25.2 mg, 0.100 mmol) was dissolved in CH_2Cl_2 (10.0 ml) inside the glovebox in a volumetric flask. This solution was diluted in a volumetric flask (200 μL in 2.00 ml). The following concentration was present: [(^{t}BuO)$_3$P] = 1.0 mM.
- (^{t}BuO)$_3$P (25.2 mg, 0.100 mmol) and C_4F_9I (34.6 mg, 0.100 mmol) were dissolved in CH_2Cl_2 (10.0 ml) inside the glovebox in a volumetric flask. This solution was diluted in a volumetric flask (200 μL in 2.00 ml). The following concentration was present: [(^{t}BuO)$_3$P] = 1 mM, [C_4F_9I] = 1 mM.

S6. Further Computational Details

All line spectra were broadened by Gaussians with standard deviation $\sigma = 1500\ \mathrm{cm}^{-1}$. The isovalue for illustrating the molecular orbitals has been set to 0.05.

S7. Absorption Spectra and Molecular Orbitals

S7.1. Perfluorobutyl Iodide

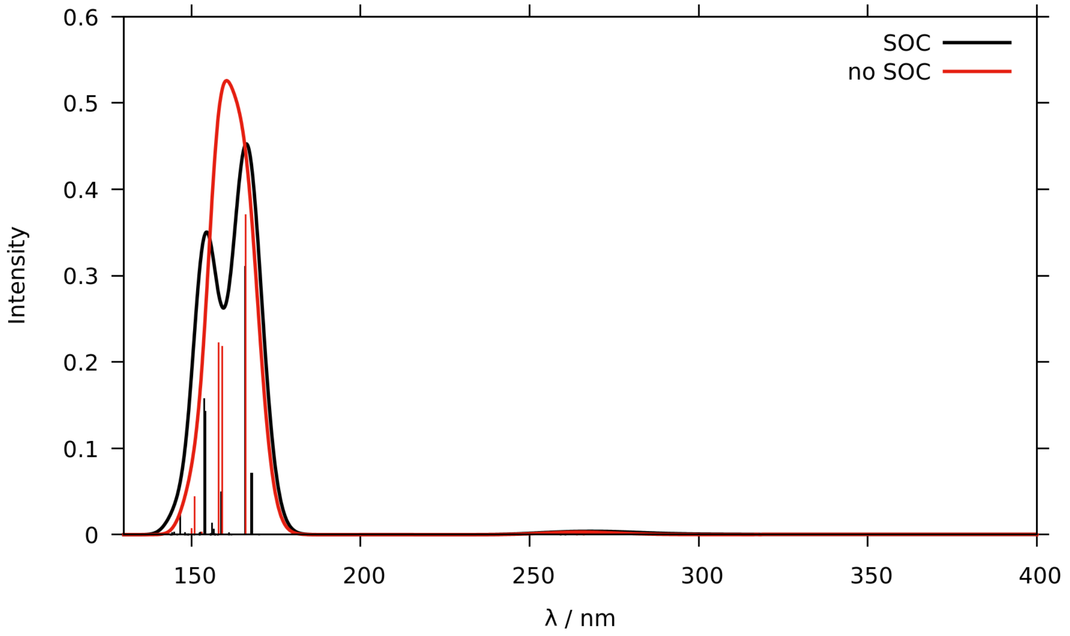

Figure S1. Calculated absorption spectra of C_4F_9I in CH_2Cl_2 with (black) and without (red) spin–orbit coupling (130 – 400 nm).

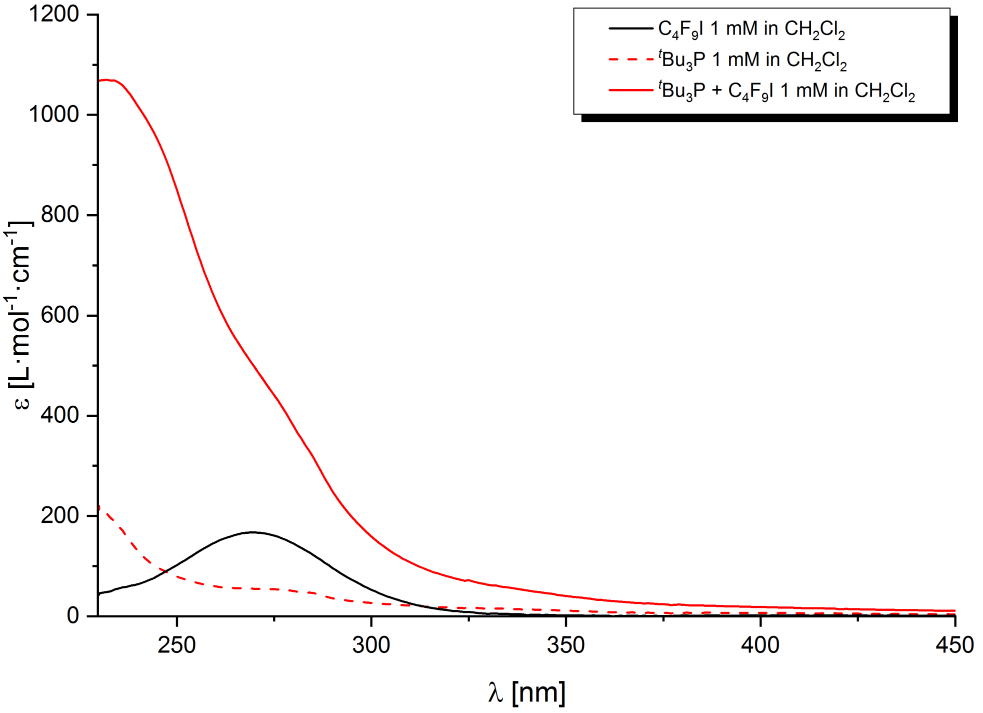

Figure S2. Experimental UV-vis spectra of C_4F_9I (λ_{max} = 270 nm), $^tBu_3P$ (λ_{max} = 227 nm) and $^tBu_3P$ + C_4F_9I (λ_{max} = 232 nm) in CH_2Cl_2.

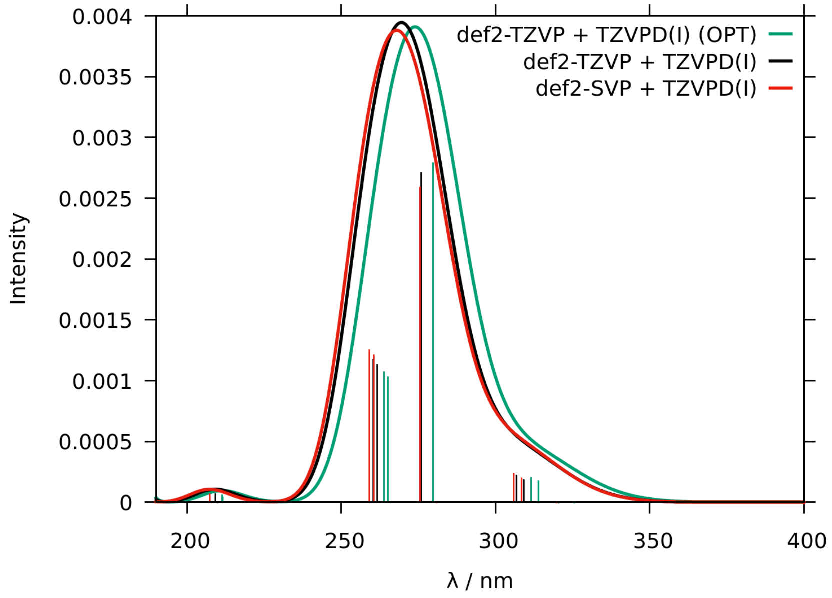

Figure S3. Atomic orbital basis set dependence of the calculated absorption spectrum of C_4F_9I (190 – 400 nm) in CH_2Cl_2 including spin–orbit coupling in quasi-degenerate perturbation theory (DFT/MRCI+SOCQDPT). The red spectrum corresponds to a calculation in the smaller def2-SVP + TZVPD(I) basis set. The black curve, labeled def2-TZVP + TZVPD(I), results from a single-point calculation using the larger def2-TZVP + TZVPD(I) basis set but employing the same geometry parameters as the red one. The green spectrum, labeled def2-TZVP + TZVPD(I) (OPT), was obtained from a set up using the larger def2-TZVP + TZVPD(I) basis set in both, the geometry optimization and DFT/MRCI+SOCQDPT step.

191 *S7.2. Phosphines and Phosphites*

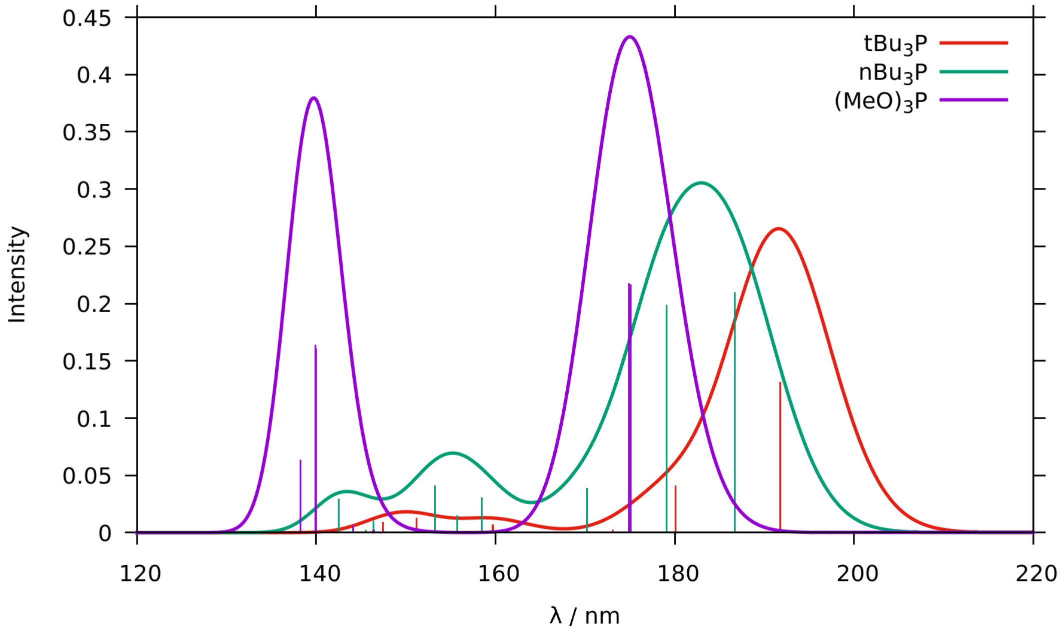

Figure S4. Computed absorption spectra of the phosphines ($^{t}Bu_3P$, $^{n}Bu_3P$) and the phosphite $(MeO)_3P$ in CH_2Cl_2 with spin–orbit coupling (120 – 220 nm).

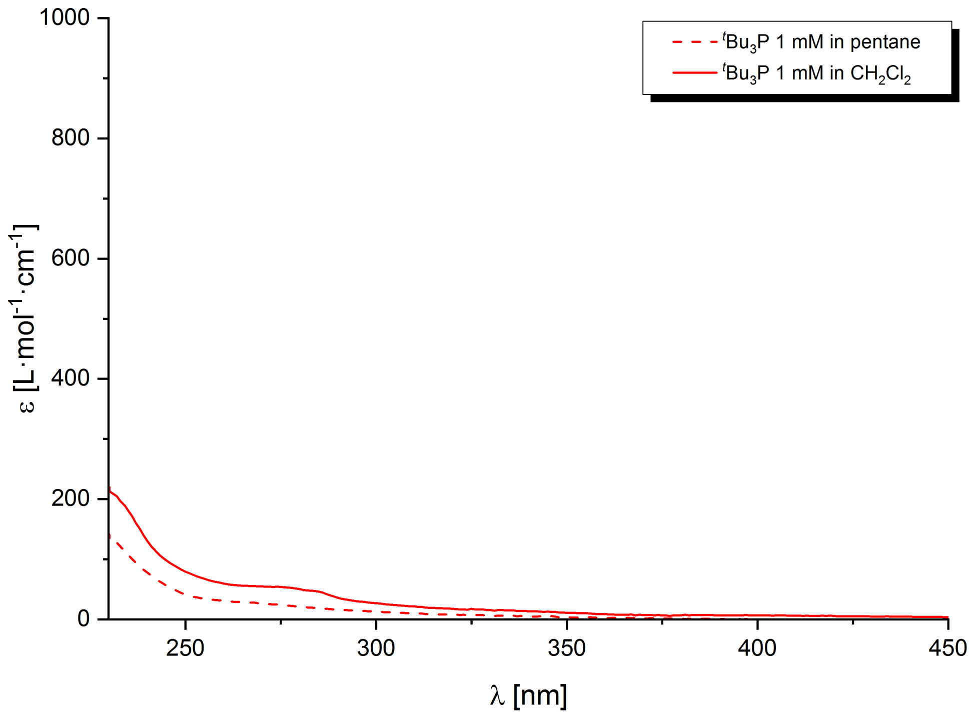

Figure S5. Comparison of the experimental UV-vis spectra of $^tBu_3P$ in pentane (λ_{max} = 227 nm) and in CH_2Cl_2 (λ_{max} = 227 nm).

192 *S7.3. Phosphine and Phosphite Adducts*

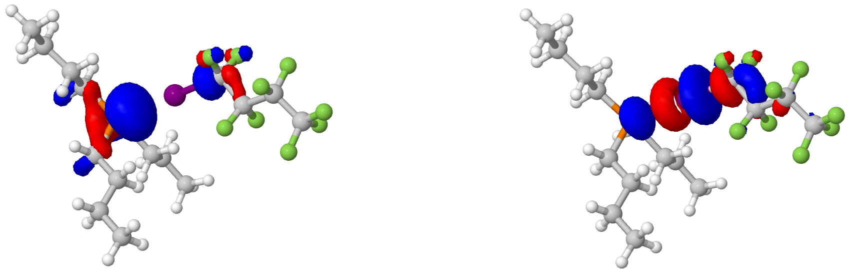

(a) HOMO: n_P (b) LUMO: σ^*

Figure S6. Frontier molecular orbitals of the $^nBu_3P$-IC_4F_9 adduct at the S_0 geometry.

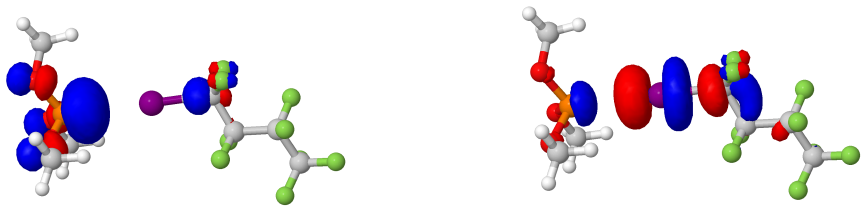

(a) HOMO: n_P (b) LUMO: σ^*

Figure S7. Frontier molecular orbitals of the $(MeO)_3P$-IC_4F_9 adduct at the S_0 geometry.

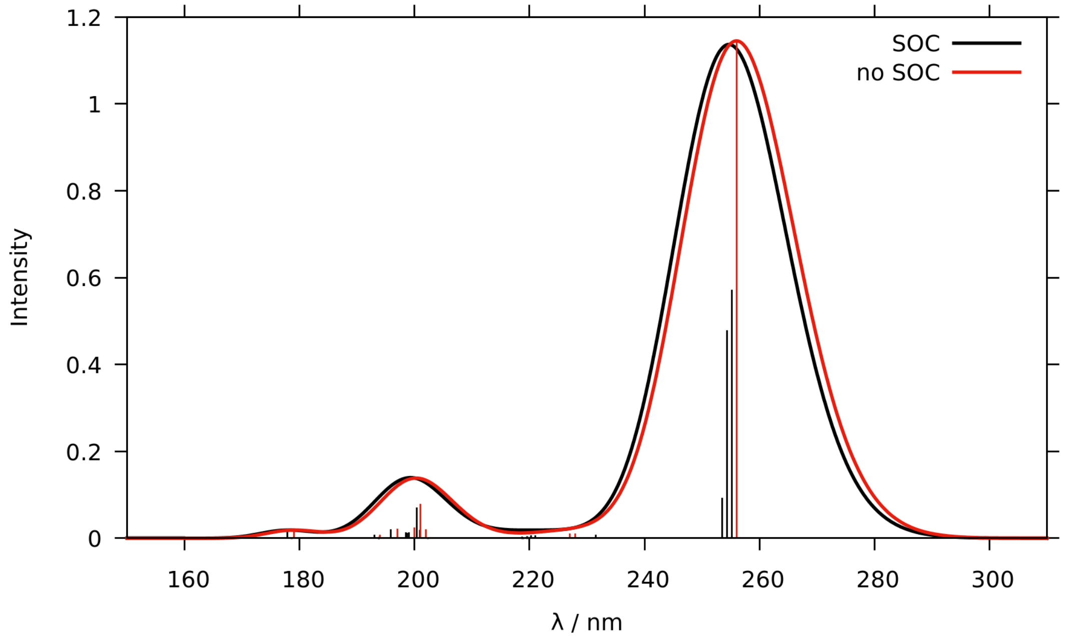

Figure S8. Calculated absorption spectra of ${}^{t}Bu_3P–C_4F_9I$ in CH_2Cl_2 with (black) and without (red) spin–orbit coupling (150 – 310 nm).

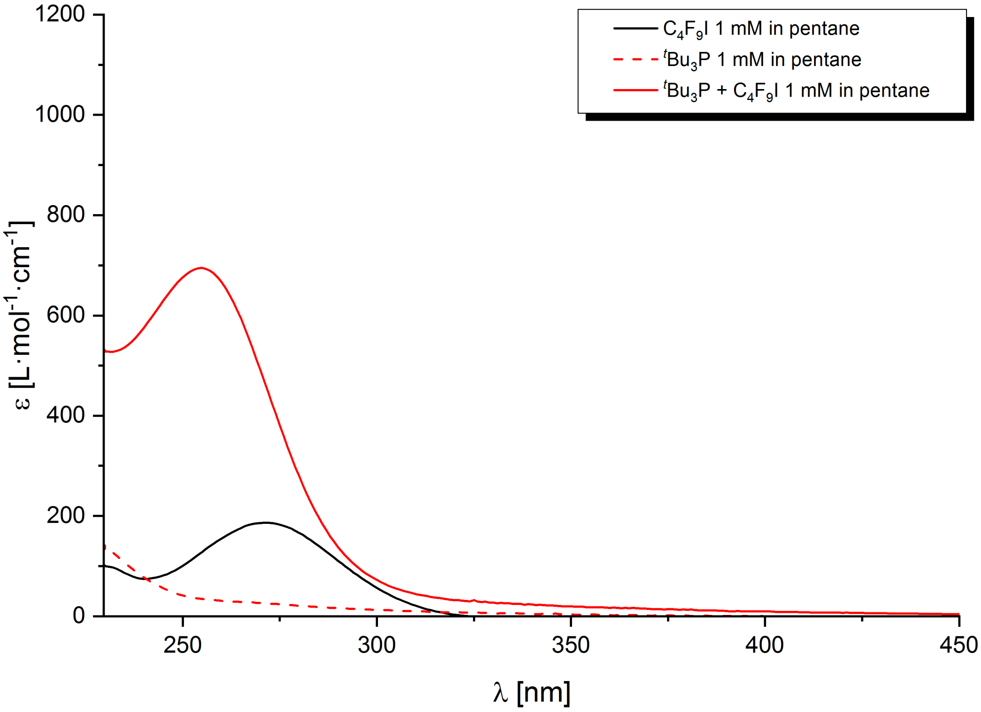

Figure S9. Experimental UV-vis spectra of C_4F_9I (λ_{max} = 270 nm), ${}^{t}Bu_3P$ (λ_{max} = 227 nm) and ${}^{t}Bu_3P$ + C_4F_9I (λ_{max} = 255 nm) in pentane.

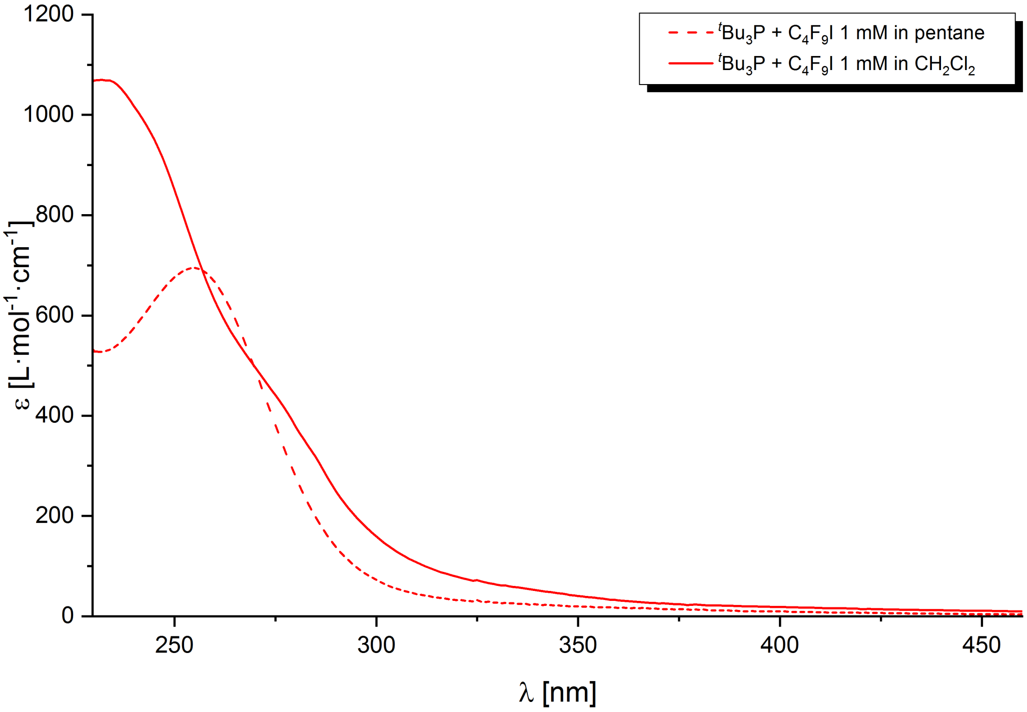

Figure S10. Comparison of the experimental UV-vis spectra of $^tBu_3P + C_4F_9I$ in pentane (λ_{max} = 255 nm) and in CH_2Cl_2 (λ_{max} = 232 nm).

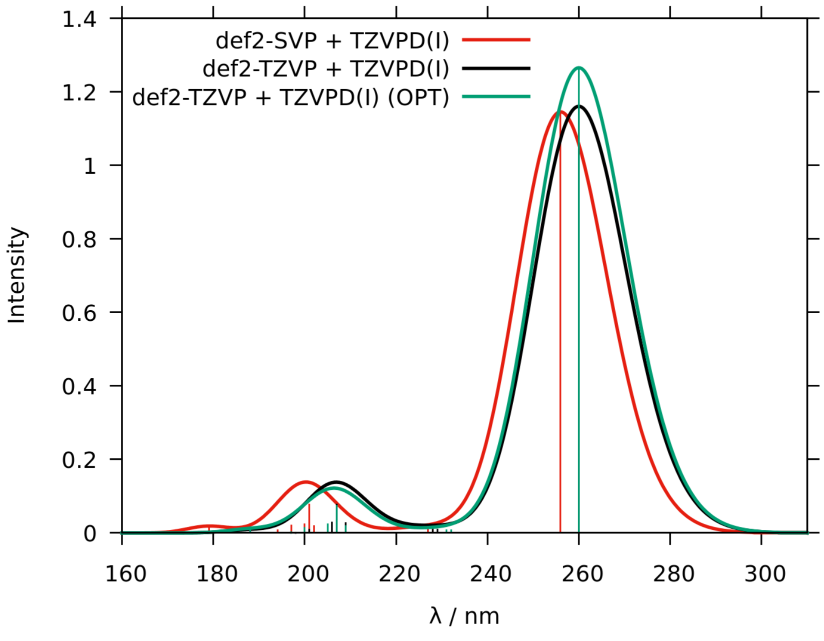

Figure S11. Atomic orbital basis set dependence of the calculated DFT/MRCI singlet absorption spectrum of the $^tBu_3P–C_4F_9I$ adduct complex (160 – 310 nm). The red spectrum corresponds to a calculation in the smaller def2-SVP + TZVPD(I) basis set. The black curve, labeled def2-TZVP + TZVPD(I), results from a single-point calculation using the larger def2-TZVP + TZVPD(I) basis set but employing the same geometry parameters as the red one. The green spectrum, labeled def2-TZVP + TZVPD(I) (OPT), was obtained from a setup using the larger def2-TZVP + TZVPD(I) basis set in both, the geometry optimization and DFT/MRCI step.

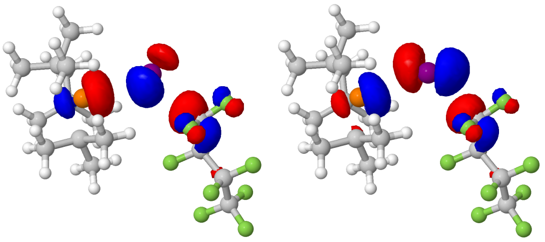

(a) ^{t}Bu$_3$P-IC$_4$F$_9$ SOMO1 (b) ^{t}Bu$_3$P-IC$_4$F$_9$ SOMO2

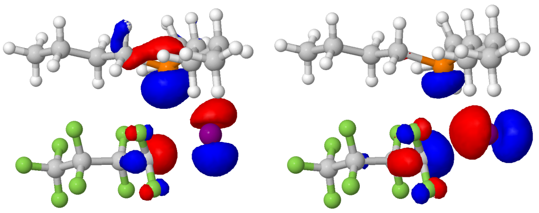

(c) ^{n}Bu$_3$P-IC$_4$F$_9$ SOMO1 (d) ^{n}Bu$_3$P-IC$_4$F$_9$ SOMO2

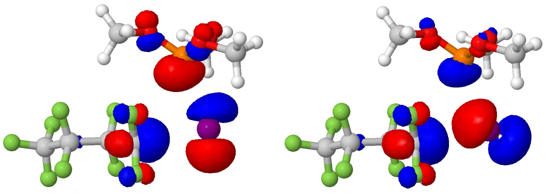

(e) (MeO)$_3$P-IC$_4$F$_9$ SOMO1 (f) (MeO)$_3$P-IC$_4$F$_9$ SOMO2

Figure S12. Singly occupied MOs (SOMOs) of the phosphine and phosphite adducts in the relaxed T_1 state.

S7.4. Solvent Influence on the Measured Absorption Spectra

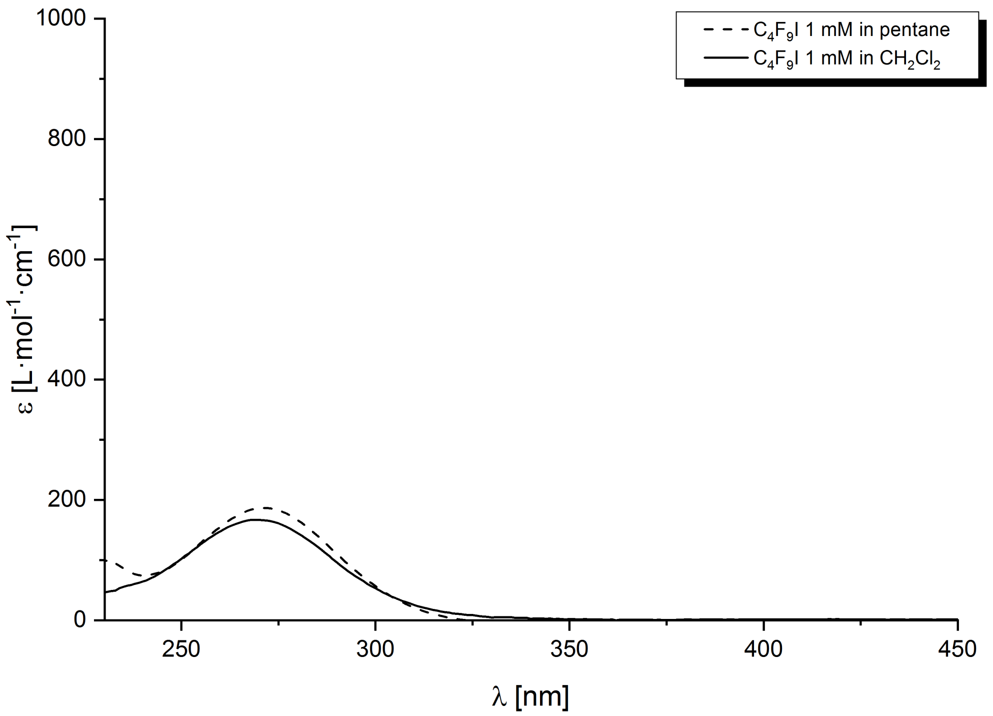

Figure S13. Comparison of the experimental UV-vis spectra of C_4F_9I in pentane (λ_{max} = 270 nm) and in CH_2Cl_2 (λ_{max} = 270 nm).

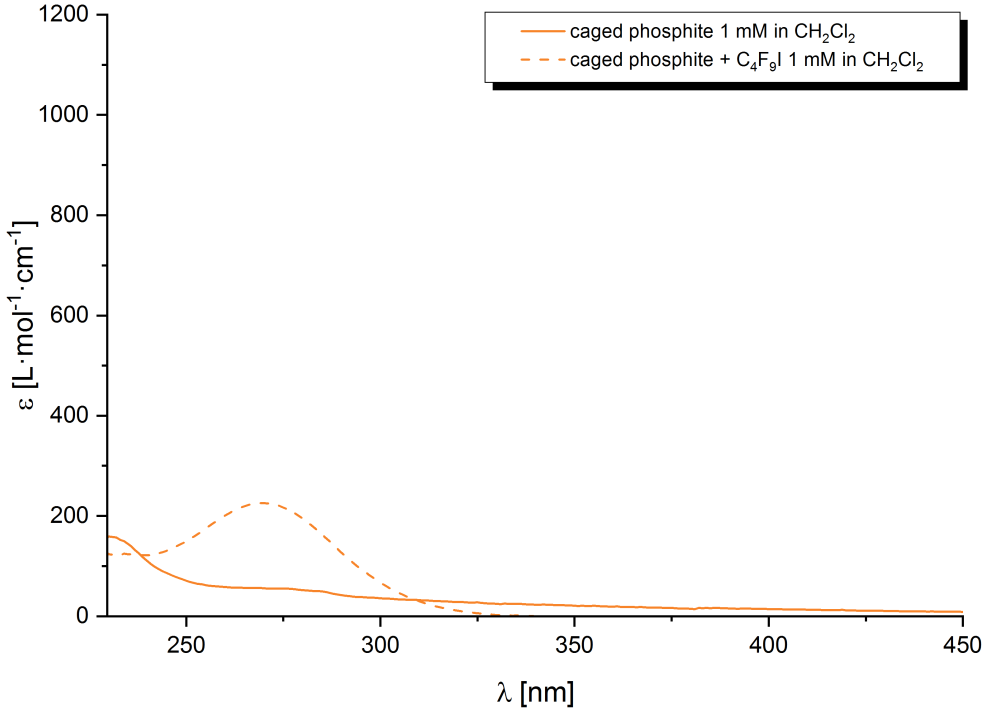

Figure S14. Comparison of the experimental UV-vis spectra of caged phosphite (λ_{max} = 230 nm) and caged phosphite + C_4F_9I (λ_{max} = 270 nm) in CH_2Cl_2.

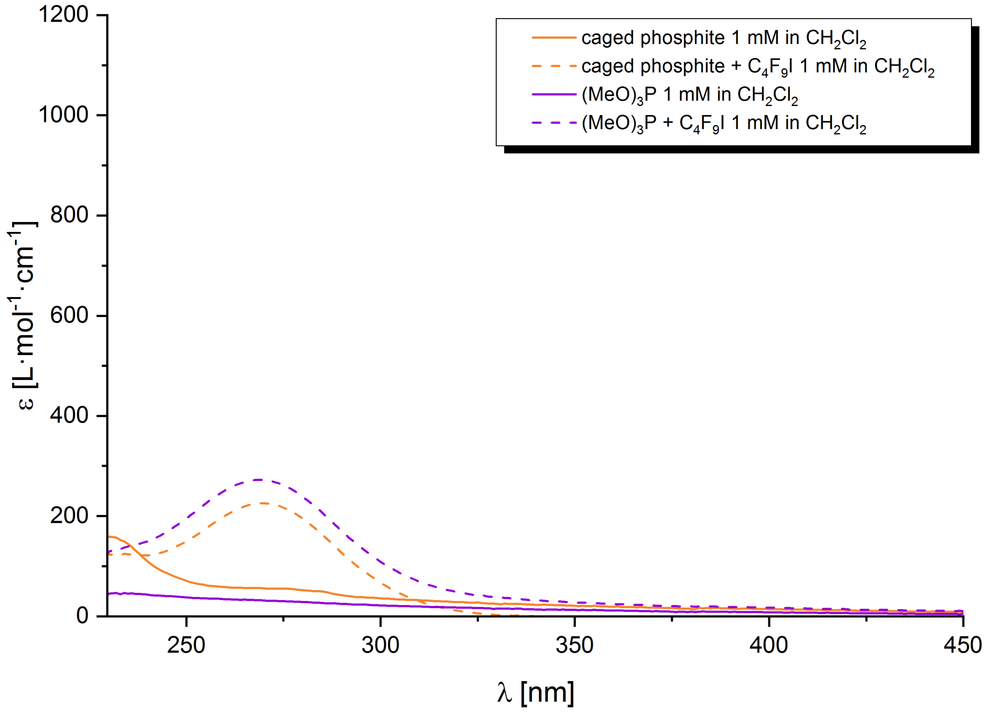

Figure S15. Comparison of the experimental UV-vis spectra of caged phosphite (λ_{max} = 230 nm), caged phosphite + C_4F_9I (λ_{max} = 270 nm), $(MeO)_3P$ ($\lambda_{max} \leq$ 230 nm) and $(MeO)_3P$ + C_4F_9I (λ_{max} = 269 nm) in CH_2Cl_2.

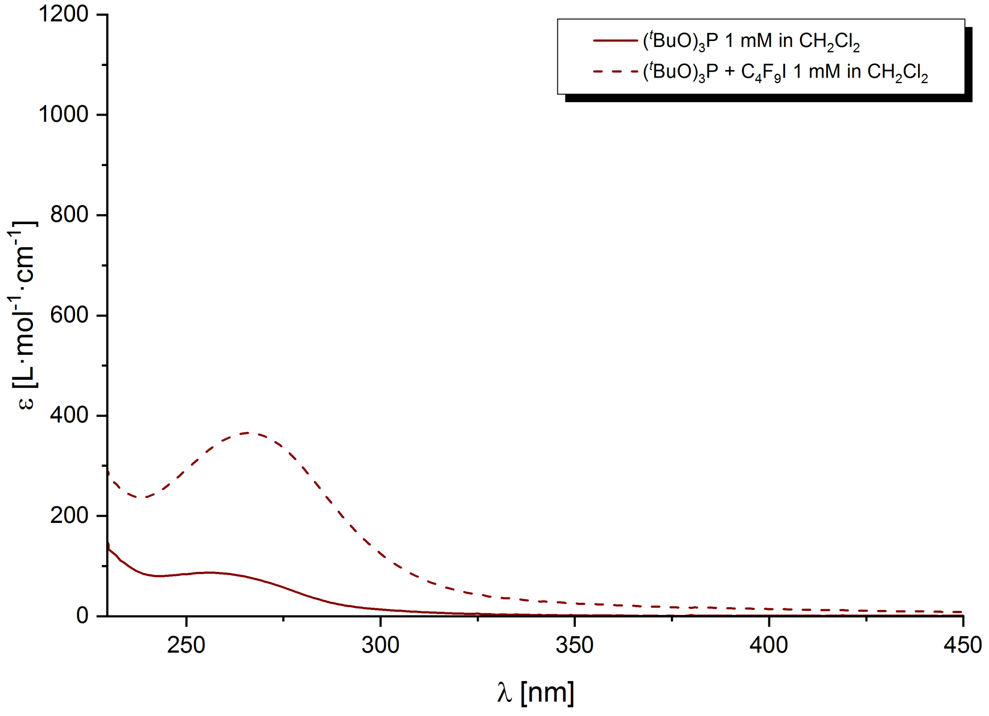

Figure S16. Comparison of the experimental UV-vis spectra of $(^tBuO)_3P$ (λ_{max} = 227 nm) and $(^tBuO)_3P$ + C_4F_9I (λ_{max} = 266 nm) in CH_2Cl_2.

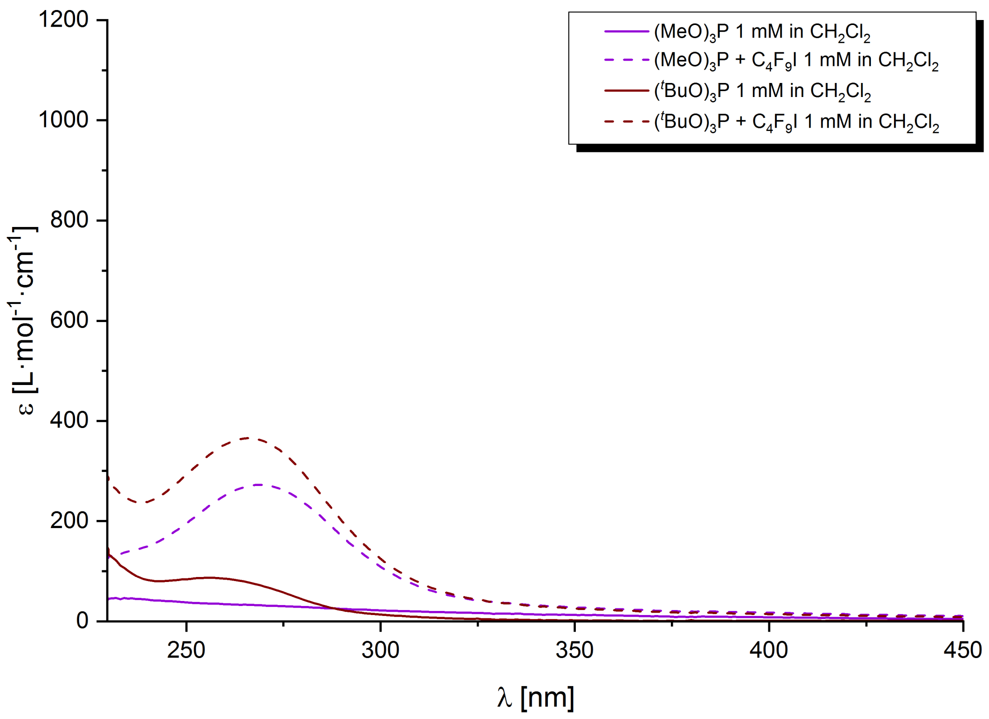

Figure S17. Comparison of the experimental UV-vis spectra of (MeO)3P ($\lambda_{max} \leq 230$ nm), (MeO)3P + C_4F_9I (λ_{max} = 269 nm), (^{t}BuO)3P (λ_{max} = 227 nm) and (^{t}BuO)3P + C_4F_9I (λ_{max} = 266 nm) in CH_2Cl_2.

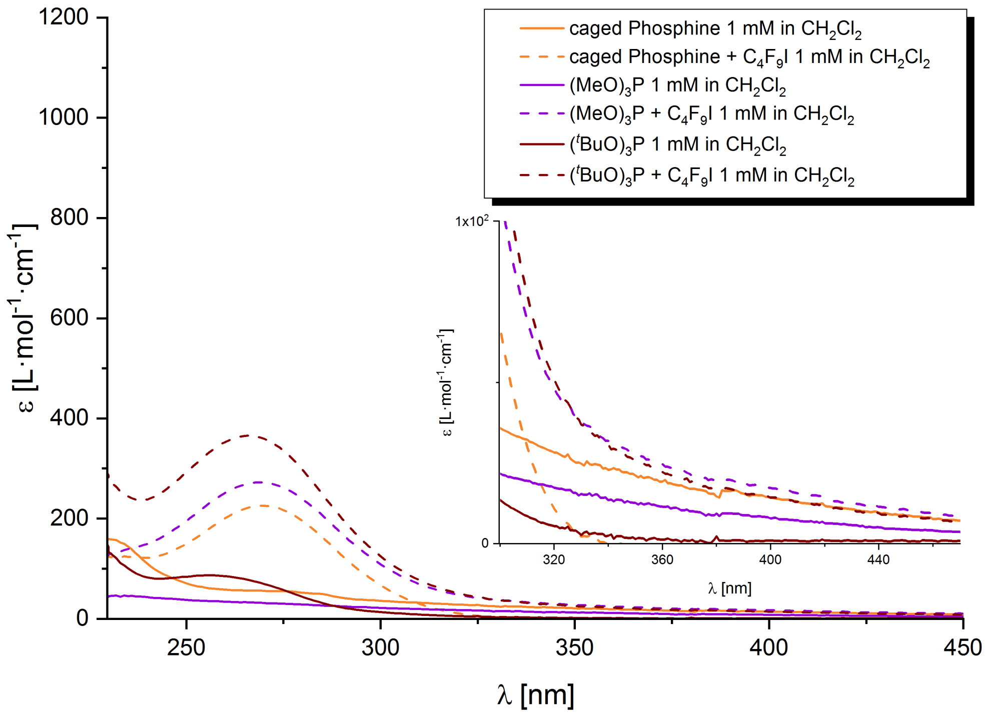

Figure S18. Comparison of the experimental UV-vis spectra of caged phosphite (λ_{max} = 230 nm), caged phosphite + C_4F_9I (λ_{max} = 270 nm), (MeO)3P ($\lambda_{max} \leq$ 230 nm), (MeO)3P + C_4F_9I (λ_{max} = 269 nm), (^{t}BuO)3P (λ_{max} = 227 nm) and (^{t}BuO)3P + C_4F_9I (λ_{max} = 266 nm) in CH_2Cl_2.

194 *S7.5. Impact of Spin–Orbit Coupling on the Calculated Spectra*

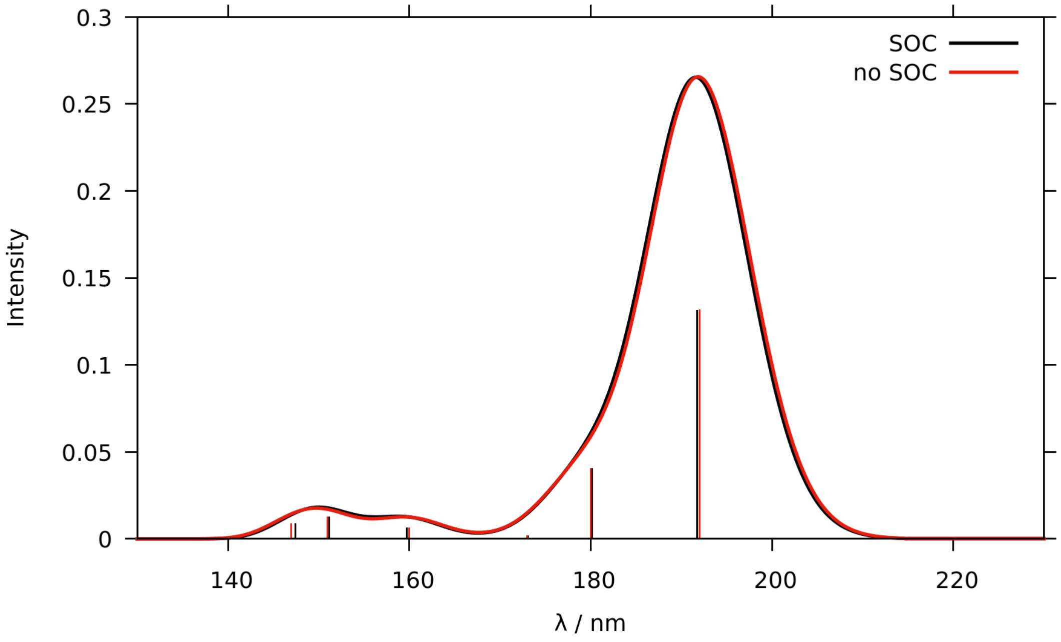

Figure S19. Calculated absorption spectra of ${}^{t}Bu_3P$ with (black) and without (red) spin–orbit coupling (130 – 230 nm).

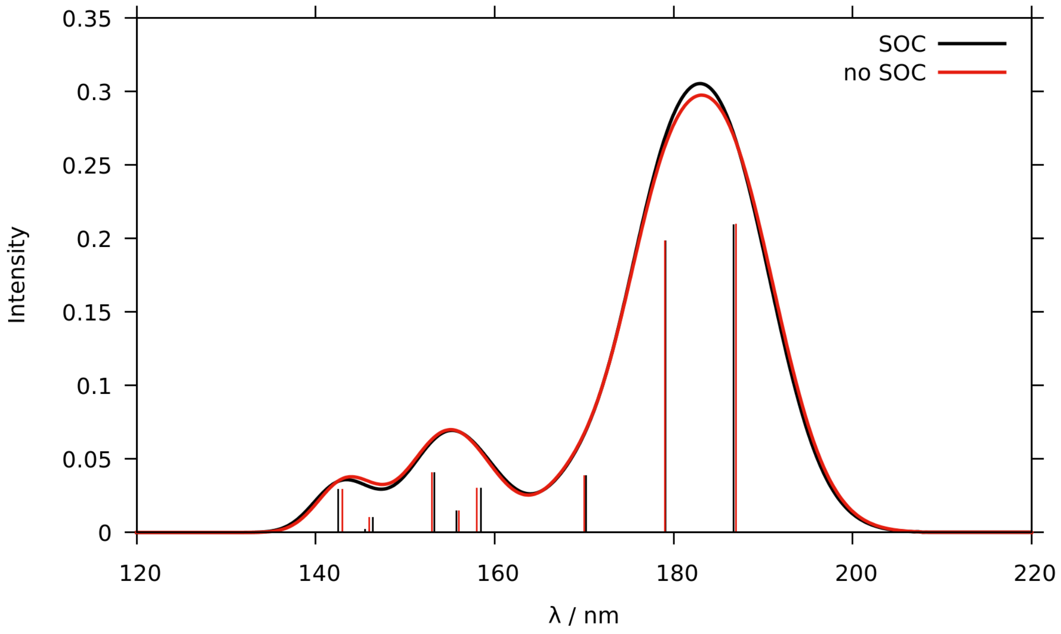

Figure S20. Calculated absorption spectra of ${}^{n}Bu_3P$ with (black) and without (red) spin–orbit coupling (120 – 220 nm).

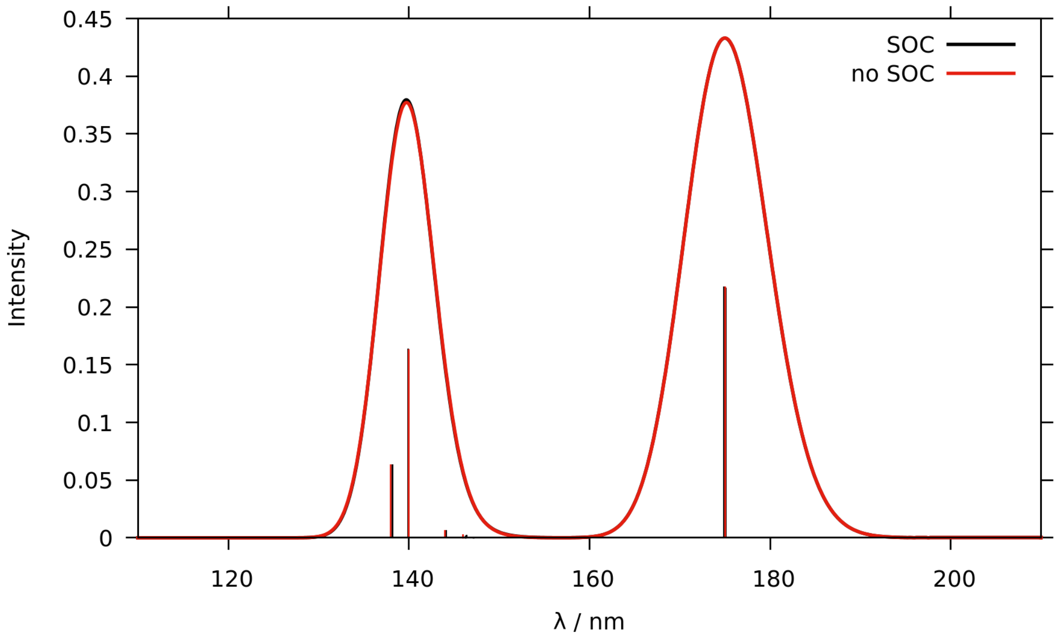

Figure S21. Calculated absorption spectra of $(MeO)_3P$ with (black) and without (red) spin–orbit coupling (110 – 210 nm).

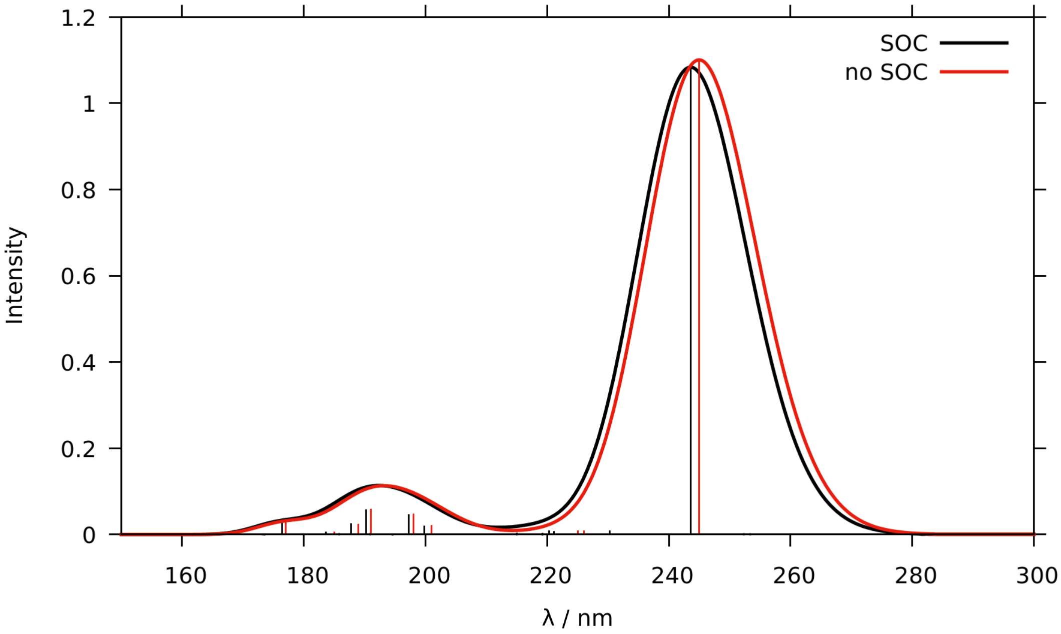

Figure S22. Calculated absorption spectra of $^{n}Bu_3P–C_4F_9I$ with (black) and without (red) spin–orbit coupling (150 – 300 nm).

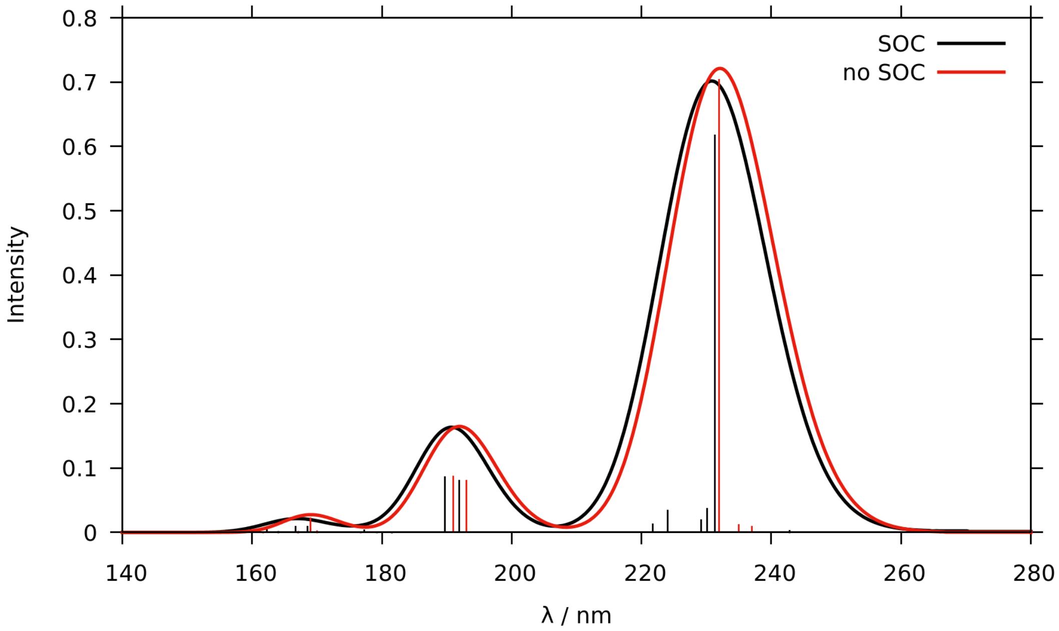

Figure S23. Calculated absorption spectra of $(MeO)_3P–C_4F_9I$ with (black) and without (red) spin–orbit coupling (140 – 280 nm).

195 ## S8. Minimum Nuclear Arrangements

196 ### *S8.1. DFT-Optimized Ground-State Geometries*

197 Perfluoroalkyl Iodide

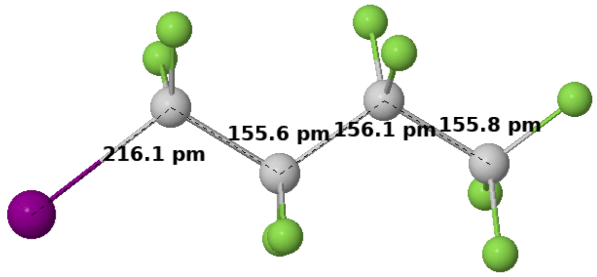

Figure S24. S_0 geometry of C_4F_9I and selected bond lengths in pm.

Phosphines

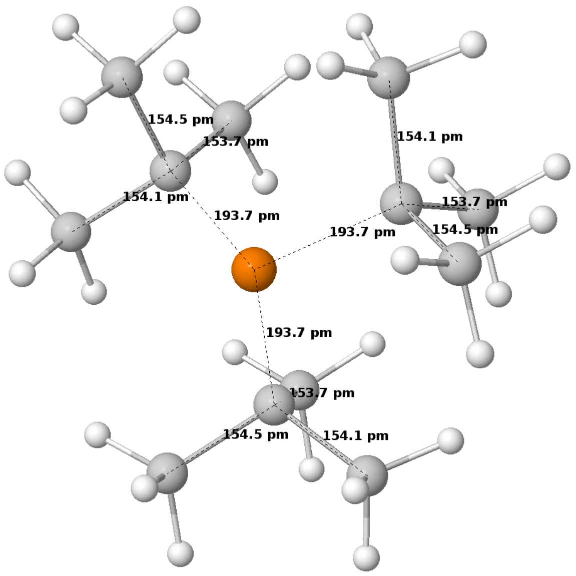

Figure S25. S_0 geometry of ^{t}Bu$_3$P and selected bond lengths in pm.

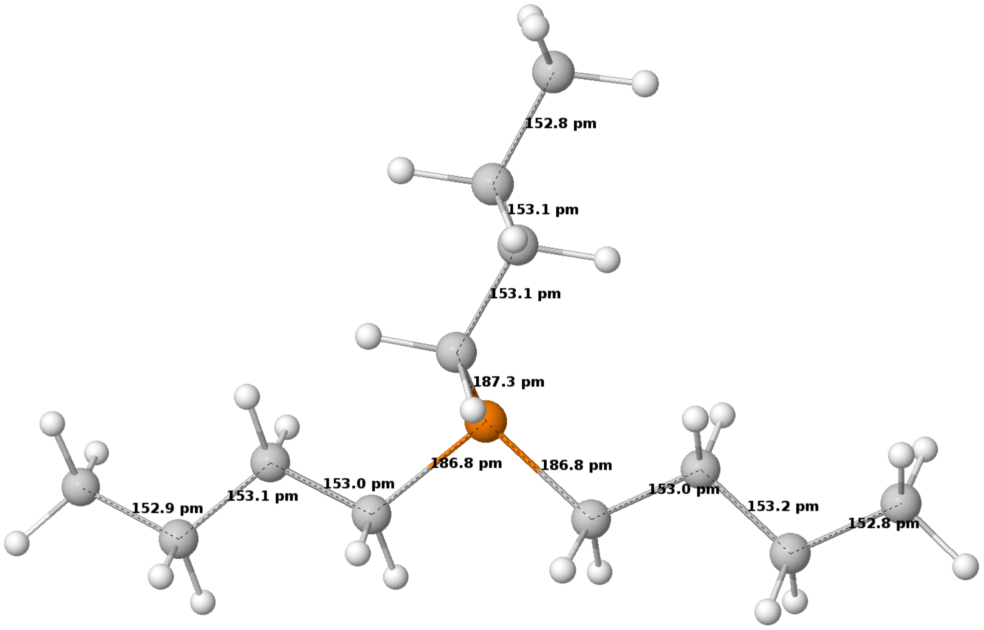

Figure S26. S_0 geometry of ^{n}Bu$_3$P and selected bond lengths in pm.

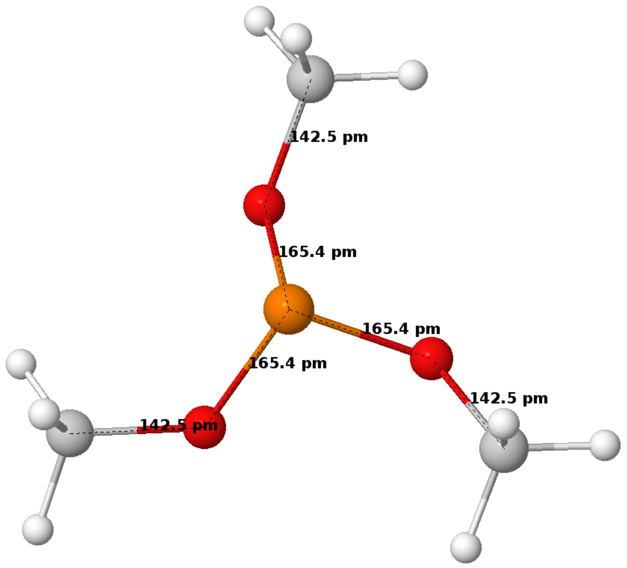

Figure S27. S_0 geometry of $(MeO)_3P$ and selected bond lengths in pm.

199 Adducts

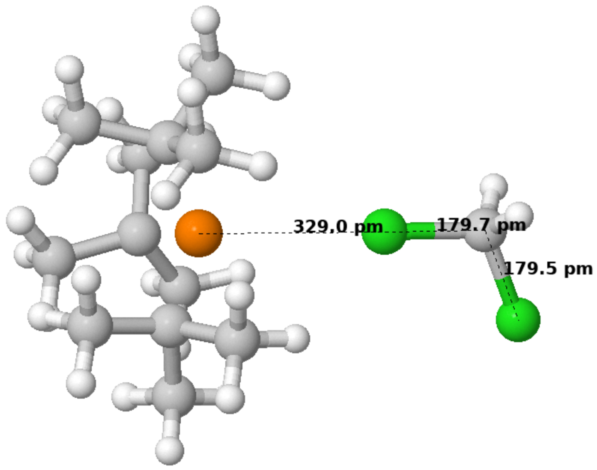

Figure S28. S_0 geometry of $^tBu_3P–CH_2Cl_2$and selected bond lengths in pm.

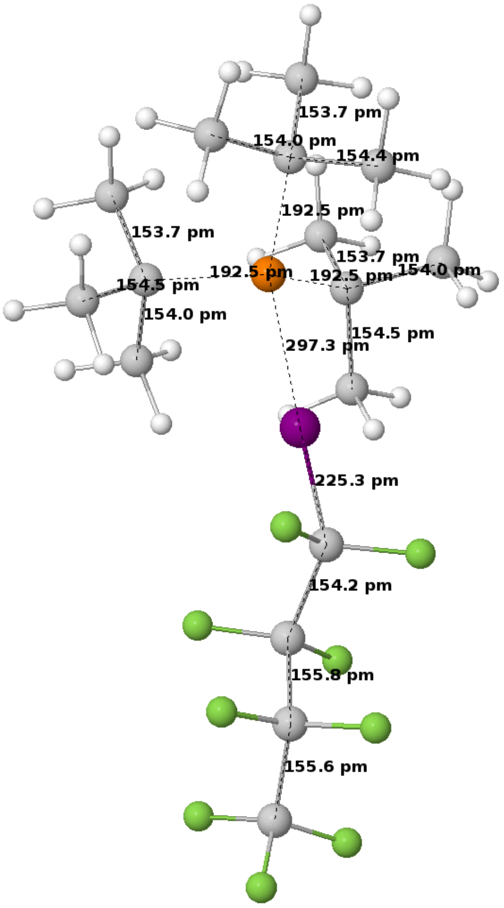

Figure S29. S_0 geometry of $^t Bu_3P–IC_4F_9$ and selected bond lengths in pm.

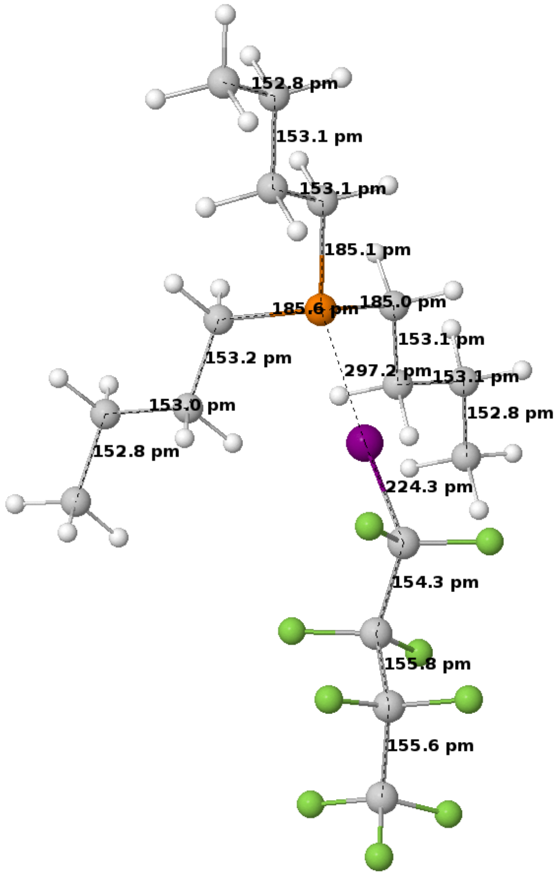

Figure S30. S_0 geometry of $^{n}Bu_3P–IC_4F_9$ and selected bond lengths in pm.

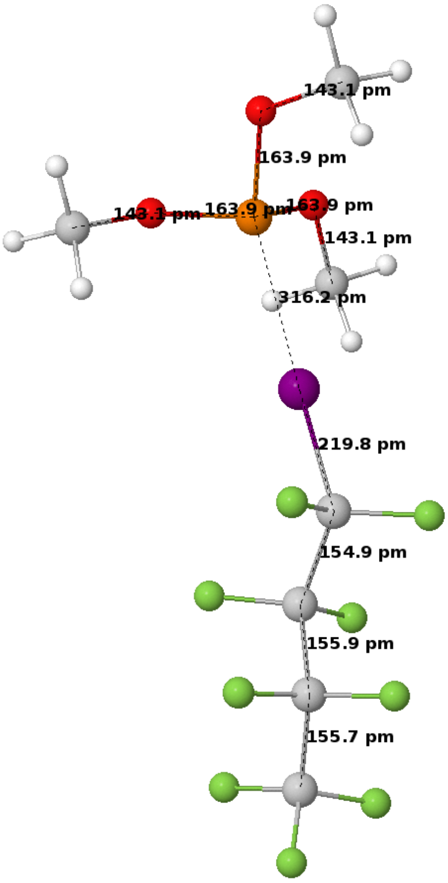

Figure S31. S_0 geometry of $(MeO)_3P–IC_4F_9$ and selected bond lengths in pm.

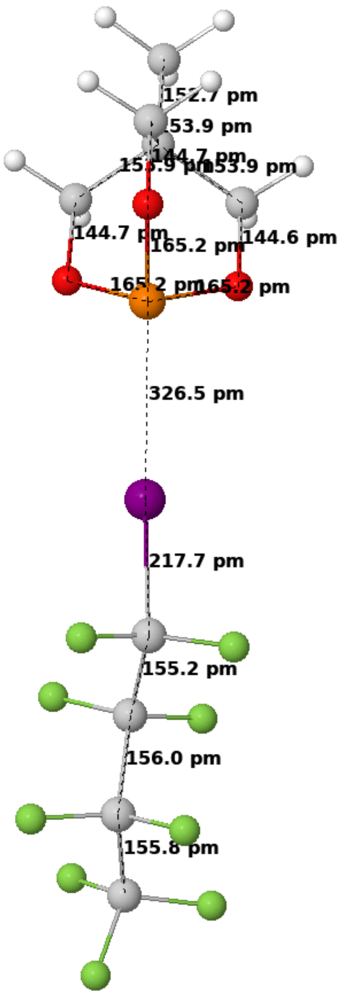

Figure S32. S_0 geometry of the caged phosphite–IC_4F_9 adduct and selected bond lengths in pm.

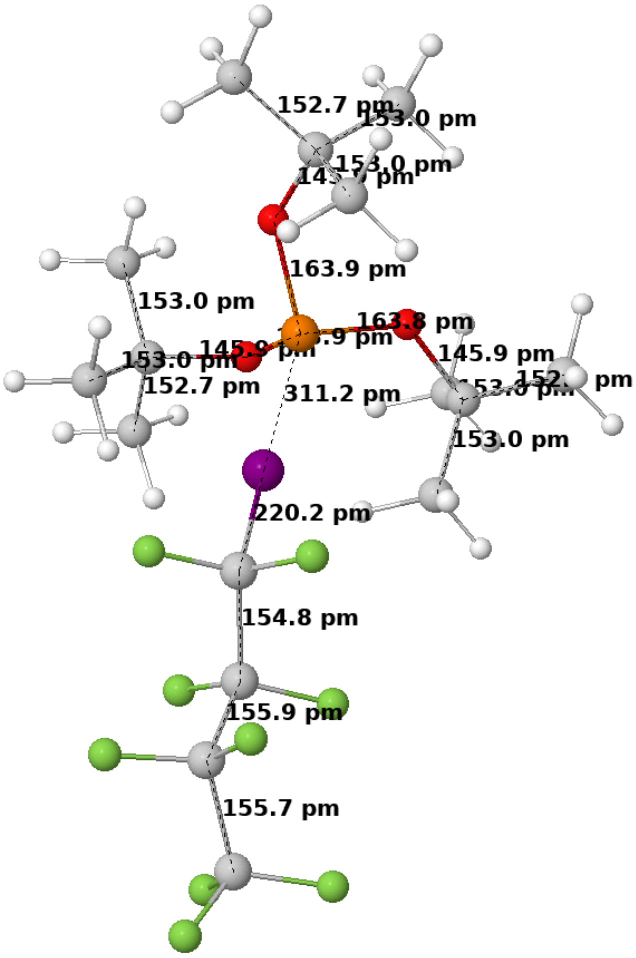

Figure S33. S_0 geometry of (^{t}BuO)$_3$P–IC_4F_9 and selected bond lengths in pm.

200 *S8.2. TDDFT/TDA-Optimized Conical Intersection Geometries*

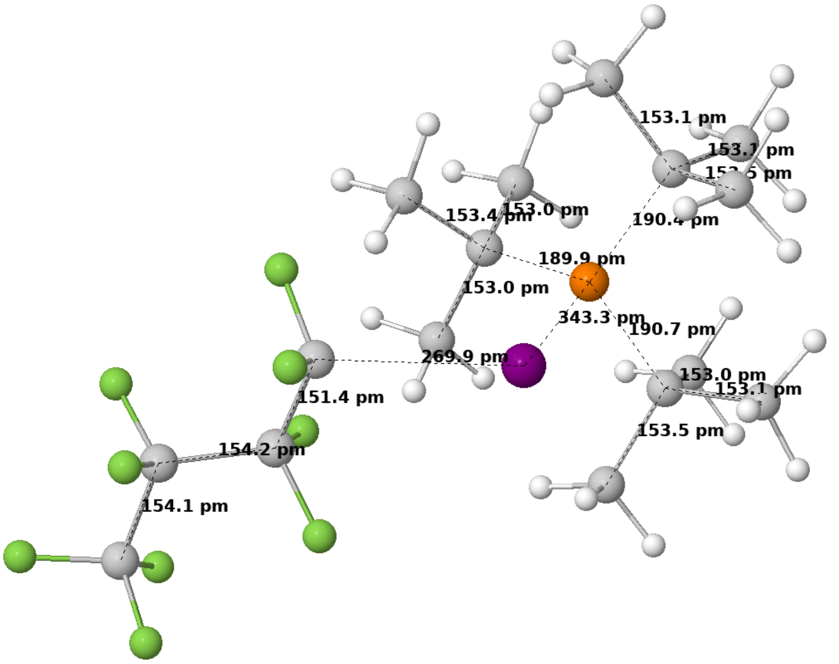

Figure S34. Geometry of the S_1/S_0 conical intersection of ^{t}Bu$_3$P–IC$_4$F$_9$ and selected bond lengths in pm.

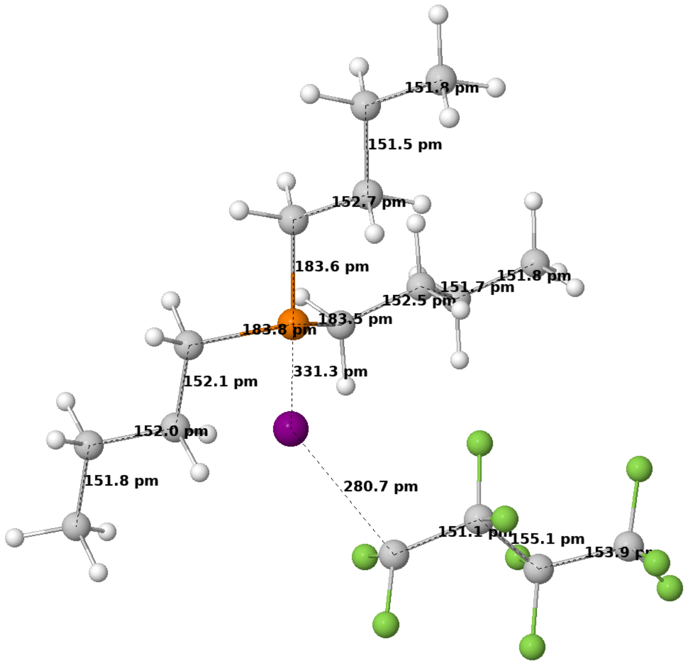

Figure S35. Geometry of the S_1/S_0 conical intersection of ^{n}Bu$_3$P–IC$_4$F$_9$ and selected bond lengths in pm.

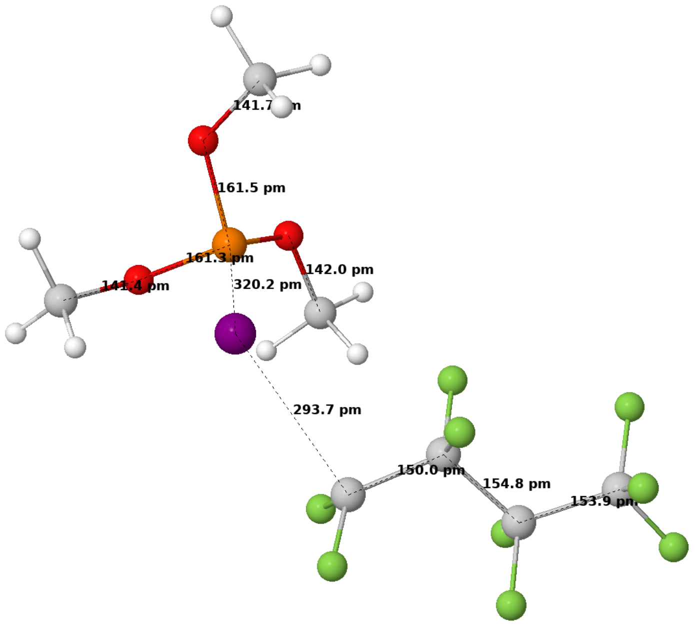

Figure S36. Geometry of the S_1/S_0 conical intersection of $(MeO)_3P–IC_4F_9$ and selected bond lengths in pm.

201 *S8.3. TDDFT/TDA-Optimized Triplet Geometries*

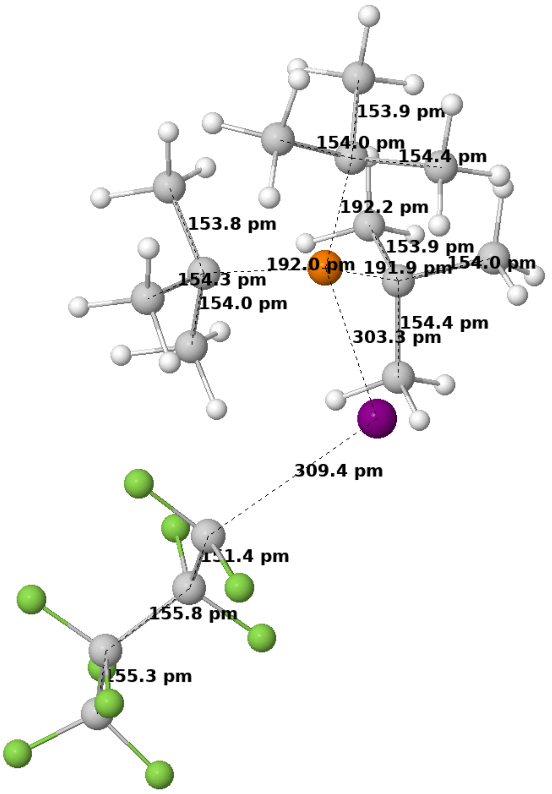

Figure S37. T_1 geometry of ${}^{t}Bu_3P–IC_4F_9$ and selected bond lengths in pm.

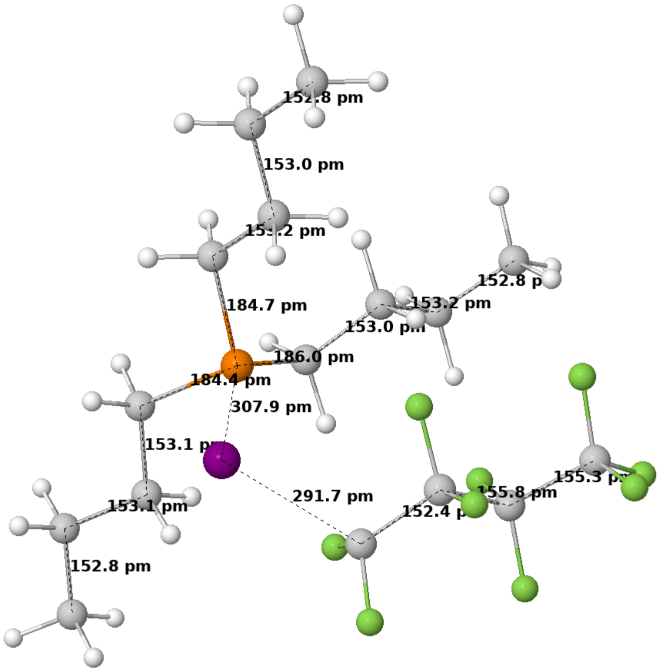

Figure S38. T_1 geometry of $^{n}Bu_3P–IC_4F_9$ and selected bond lengths in pm.

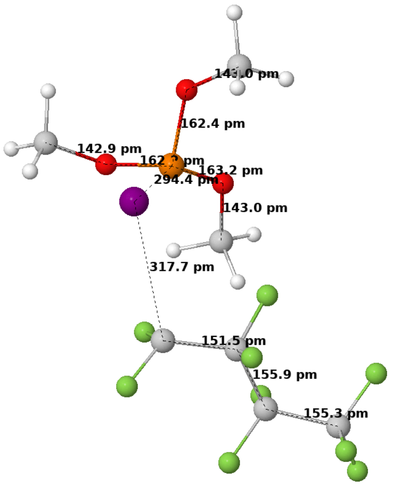

Figure S39. T_1 geometry of $(MeO)_3P–IC_4F_9$ and selected bond lengths in pm.

1. Helmecke, L.; Spittler, M.; Baumgarten, K.; Czekelius, C. Metal-Free Activation of C–I Bonds and Perfluoroalkylation of Alkenes with Visible Light Using Phosphine Catalysts. *Org. Lett.* **2019**, *21*, 7823–7827. doi:10.1021/acs.orglett.9b02812.
2. Cole, J.R.; Dellinger, M.E.; Johnson, T.J.; Reinecke, B.A.; Pike, R.D.; Pennington, W.T.; Krawiec, M.; Rheingold, A.L. Caged phosphite complexes of copper(I) halides. *J. Chem. Crystallogr.* **2003**, *33*, 341–347. doi:10.1023/A:1024217727932.
3. Taira, K.; Mock, W.L.; Gorenstein, D.G. Experimental Tests of the Stereoelectronic Effect at Phosphorus: Nucleophilic Reactivity of Phosphite Esters. *J. Am. Chem. Soc.* **1984**, *106*, 7831–7835. doi:10.1021/ja00337a029.
4. Manning, H.C.; Bai, M.; Anderson, B.M.; Lisiak, R.; Samuelson, L.E.; Bornhop, D.J. Expeditious synthesis of 'P'-protected macrocycles en route to lanthanide chelate metal complexes. *Tetrahedron Lett.* **2005**, *46*, 4707–4710. doi:10.1016/j.tetlet.2005.05.049.
5. Bietti, M.; Calcagni, A.; Salamone, M. The Role of Structural Effects on the Reactions of Alkoxyl Radicals with Trialkyl and Triaryl Phosphites. A Time-Resolved Kinetic Study. *J. Org. Chem.* **2010**, *75*, 4514–4520. doi:10.1021/jo100703b.
6. Mark, V.; Wazer, J.R.V. Tri-*t*-butyl Phosphite and Some of Its Reactions. *J. Org. Chem.* **1964**, *29*, 1006–1008. doi:10.1021/jo01028a005.
